# Life Detection and Microbial Biomarker Profiling with Signs of Life Detector-Life Detector Chip During a Mars Drilling Simulation Campaign in the Hyperarid Core of the Atacama Desert

**DOI:** 10.1089/ast.2021.0174

**Published:** 2023-12-20

**Authors:** Mercedes Moreno-Paz, Rita Sofia dos Santos Severino, Laura Sánchez-García, Juan Manuel Manchado, Miriam García-Villadangos, Jacobo Aguirre, Miguel Angel Fernández-Martínez, Daniel Carrizo, Linda Kobayashi, Arwen Dave, Kim Warren-Rhodes, Alfonso Davila, Carol R. Stoker, Brian Glass, Víctor Parro

**Affiliations:** ^1^ Department of Molecular Evolution, Centro de Astrobiología (CAB), INTA-CSIC, Madrid, Spain.; ^2^ Departament of Física y Matemáticas y de Automática, University of Alcalá de Henares (UAH), Madrid, Spain.; ^3^ Department of Natural Resource Sciences, McGill University, Québec, Canada.; ^4^ Space Science Division and Astrobiology Division, NASA Ames Research Center, Moffett Field, California, USA.; ^5^ Carl Sagan Center, SETI Institute, Mountain View, California, USA.

**Keywords:** Biomarkers, Atacama Desert, Planetary exploration, SOLID-LDChip, Metaproteomics

## Abstract

The low organic matter content in the hyperarid core of the Atacama Desert, together with abrupt temperature shifts and high ultraviolet radiation at its surface, makes this region one of the best terrestrial analogs of Mars and one of the best scenarios for testing instrumentation devoted to *in situ* planetary exploration. We have operated remotely and autonomously the SOLID-LDChip (Signs of Life Detector-Life Detector Chip), an antibody microarray-based sensor instrument, as part of a rover payload during the 2019 NASA Atacama Rover Astrobiology Drilling Studies (ARADS) Mars drilling simulation campaign. A robotic arm collected drilled cuttings down to 80 cm depth and loaded SOLID to process and assay them with LDChip for searching for molecular biomarkers. A remote science team received and analyzed telemetry data and LDChip results. The data revealed the presence of microbial markers from Proteobacteria, Acidobacteria, Bacteroidetes, Actinobacteria, Firmicutes, and Cyanobacteria to be relatively more abundant in the middle layer (40–50 cm). In addition, the detection of several proteins from nitrogen metabolism indicates a pivotal role in the system. These findings were corroborated and complemented on “returned samples” to the lab by a comprehensive analysis that included DNA sequencing, metaproteomics, and a metabolic reconstruction of the sampled area. Altogether, the results describe a relatively complex microbial community with members capable of nitrogen fixation and denitrification, sulfur oxidation and reduction, or triggering oxidative stress responses, among other traits. This remote operation demonstrated the high maturity of SOLID-LDChip as a powerful tool for remote *in situ* life detection for future missions in the Solar System.

## 1. Introduction

One of future spaceflight missions' main goals will be to search for evidence of life in other habitable environments in our Solar System. Mars is one of the priority targets, because multiple lines of evidence point to a protracted period of habitable conditions early in the history of the planet (Fassett and Head, 2008, 2011; Fairén, 2010; Carter *et al*., 2015). The discovery of organic compounds such as chlorobenzene and thiophene in sediments deposited at Gale Crater during this early period (Freissinet *et al.*, 2015; Eigenbrode *et al*., 2018) motivates the search for, and characterization of, organic matter and possible molecular biomarkers in other regions that might have experienced more benign and possibly habitable conditions in more recent times.

Recent habitable conditions near the surface of Mars could have been triggered by changes in the planet's obliquity, eccentricity, and phase of perihelion (Laskar *et al*., 2002), which could result in wide variations over time of the amount of solar insolation reaching the surface. This, in turn, can increase surface temperatures in polar regions and result in high latitude ice redistribution to lower latitudes (Head *et al.*, 2003).

During high obliquity periods (the last of which occurred 5 Myr ago), near-surface ground ice could experience temperatures close to the melting point, which could lead to consideration that Mars is periodically habitable (Stoker *et al*., 2010). It has been hypothesized that, during such climate excursions, putative dormant microorganisms near the surface could take advantage of the higher temperature and moisture to become active and grow while remaining sheltered from cosmic and ultraviolet (UV) radiation. Remnants of these organisms might still be found near the surface even after several million years due to the low temperature and extreme dryness that promotes biosignature preservation.

The Icebreaker mission concept has been developed to test this hypothesis. Icebreaker features a lander with a rotary-percussive drill that can penetrate into and sample ice-cemented regolith and several life-detection instruments with which to search for molecular evidence of life (McKay *et al.*, 2013; Zacny *et al*., 2013), including the SOLID (Signs of Life Detector) instrument.

SOLID extracts organic compounds from regolith, introduces them into a liquid solvent, and searches the liquid extract for complex organic polymers with antigen-antibody reactions in a biosensor element called the LDChip (Life Detector Chip) (Rivas *et al.*, 2008; Parro *et al.*, 2011b; Moreno-Paz *et al.*, 2018; Sánchez-García *et al*., 2020). Antibody/protein microarray technology is a robust and reliable analytical approach that allows the detection of hundreds of microbial molecular biomarkers at once (Rivas *et al*., 2008).

To date, several studies have validated the use of bio-affinity sensing components such as antibody-antigen microarrays to search for evidence of life (Derveni *et al.*, 2012; Carr *et al*., 2013; Baqué *et al*., 2017; Coussot *et al*., 2019) even after the target compounds and the sensing components (antibodies) have been exposed to levels of gamma radiation higher than expected during a mission to Mars (de Diego-Castilla *et al*., 2011; Blanco *et al*., 2018). In addition, experiments outside the International Space Station have demonstrated that antibodies remain viable after exposure to a dose of ionizing radiation equivalent to the absorbed dose expected during a Mars mission (Coussot *et al*., 2019).

To verify the robustness and maturity of any instrumentation designed for space missions, it is essential to test them in relevant environments. The low levels of biomass (10^3^–10^5^ cell/g soil) and organic carbon (<100 μg/g soil) in surface soils of the hyperarid core of the Atacama Desert (Navarro-González *et al*., 2003; Valdivia-Silva *et al*., 2016) make it a good terrestrial analog for instrument testing.

**FIG. 1.**
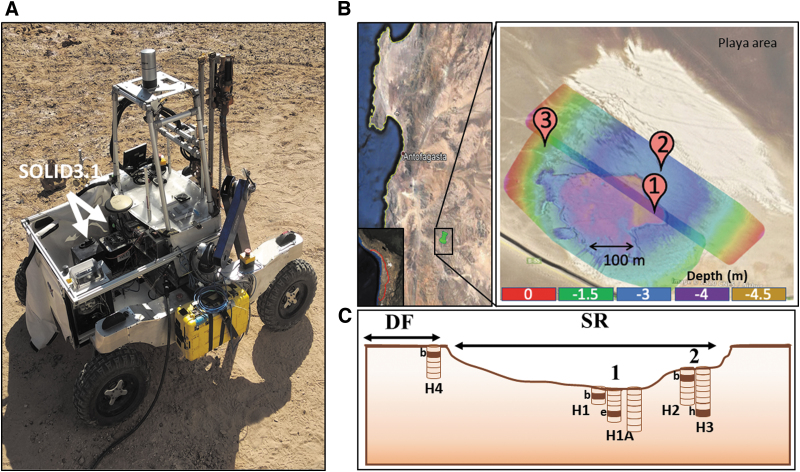
The ARADS 2019 remote Mars drilling simulation campaign in the Atacama Desert. **(A)** SOLID3.1 instrument on the autonomous K-REX2 rover at the Playa area under study. **(B)** A Google Earth image of the Playa area showing the relative position of the Playa area in a schematic topography of the field. The color code represents the elevation model from drone imaging of the field area in meters from the desert pavement. Three sections were drilled in the Playa area, corresponding to SR sediments (SR1 and SR2) and DF (3) sediments, all located below a baseline reference in the desert pavement. **(C)** A cross-section view of the Playa area showing the relative position of perforations in the three sampling locations and the spatial position and proximity between them. Samples were taken at 10 cm intervals to perform the immunoprofile with LDChip for holes H1, H1A, H2, H3 and H4 as indicated in Section 2. A subset of five samples highlighted in dark brown and labeled with a letter to indicate the depth (b for 10–20 cm, e for 40–50 cm, h for 70–80 cm) were delivered to the instruments as part of the payload of the K-REX2 rover to perform remote analyses of biomarkers and returned to the lab for subsequent analyses. ARADS, Atacama Rover Astrobiology Drilling Studies; DF, desert floor; LDChip, Life Detector Chip; SOLID, Signs of Life Detector; SR, sulfate-rich.

The ARADS, a NASA PSTAR project (Glass *et al*., 2018), was conceived for maturing and testing instrumentation and future *in situ* exploration. The 2019 ARADS field campaign conducted a field experiment with a fully integrated *in situ* sampling and analysis system, using the K-REX2 rover that carried a 1 m drill and a robotic arm to deliver the sample to onboard instruments, including SOLID3.1 (Fig. 1).

The entire payload was remotely operated, including drilling, sampling, sample delivery to SOLID, and LDChip analysis. Telemetry and analysis data were received daily by a remote science and operations team. Herein, we report the remote and *in situ* SOLID performance, science and technical findings, and ground truthing with a comprehensive set of analytical techniques in the laboratory.

## 2. Materials and Methods

### 2.1. Field site and sampling site selection

The remote science and operations team selected an evaporitic environment in one of the driest regions in the Atacama Desert (24°6′5.63″S, 70°8′15.72″W; 1013 m.a.s.l.) as the site for science operations. The campaign was carried out in September 2019.

Drill samples were obtained at three different sites in an evaporitic playa situated at the terminus of a well-drained Mio-Pliocene alluvial fan located about 20 km west of the Yungay research station (Fig. 1A, B). Two of the drill sites (SR1 and SR2) were inside a desiccated playa infilled with clay sediments (SR2 was perched c.a. 1.5 m above SR1). The third site (desert floor [DF]) was outside the playa and consisted of desert regolith (Fig. 1B, C).

The constraints of the iterative sample extraction using an autonomous drill in a remote setting can sometimes make it difficult to recover pristine samples at different depths from the same drillhole, because hole collapse and mechanical particle scattering are conducive to cross-contamination between sampling depths. Therefore, multiple, adjacent drill profiles (spaced 20–50 cm) were obtained at SR1 (labeled H1, H1A, and H5) and SR2 (labeled H2 and H3). One drill profile was obtained at DF (labeled H4). Bulk samples (10–20 g) were robotically acquired from each drillhole at 10 cm intervals.

Five bulk samples selected from all the drillholes were subsampled (c.a. 1 g) and designated for autonomous analysis with SOLID (Fig. 1C): one subsample from holes H1 at 10–20 cm (S-H1b), hole H1A at 40–50 cm (S-H1Ae), hole H2 at 10–20 cm (S-H2b), hole H3 at 70–80 cm (S-H3h), and hole H4 at 10–20 cm (S-H4b). Once loaded with the subsample, SOLID was remotely operated. The remaining bulk samples from each depth were aseptically and manually sampled in Sterile Whirl-Pak bags (c.a. 2–3 g) and transported to the field lab for ground-truth analyses. Once in the field lab, two replicates (c.a. 0.5 g) from each bulk sample were collected to perform manual fluorescence sandwich microarray immunoassays (FSMI) while in the field, using a MultiArray Analysis Module (MAAM) (Fernández-Calvo *et al*., 2006).

The remaining samples were stored at room temperature and returned to the Centro de Astrobiología laboratory in Madrid (Spain) for an additional comparative study. Only sample S-H3h was depleted after analysis in SOLID, and an alternative sample, S-H3d (30–40 cm), was used in subsequent analysis to obtain a complete depth profile.

### 2.2. Autonomous SOLID3.1 measurements

The SOLID instrument was designed to analyze solid (ice, dust, or ground rock) or liquid samples autonomously and remotely. Several SOLID prototypes have been developed and successfully tested in different field campaigns (Parro *et al*., 2005b, 2008, 2011b, 2018; Stoker *et al*., 2008; Blanco *et al*., 2012; Sánchez-García *et al*., 2020).

**FIG. 2.**
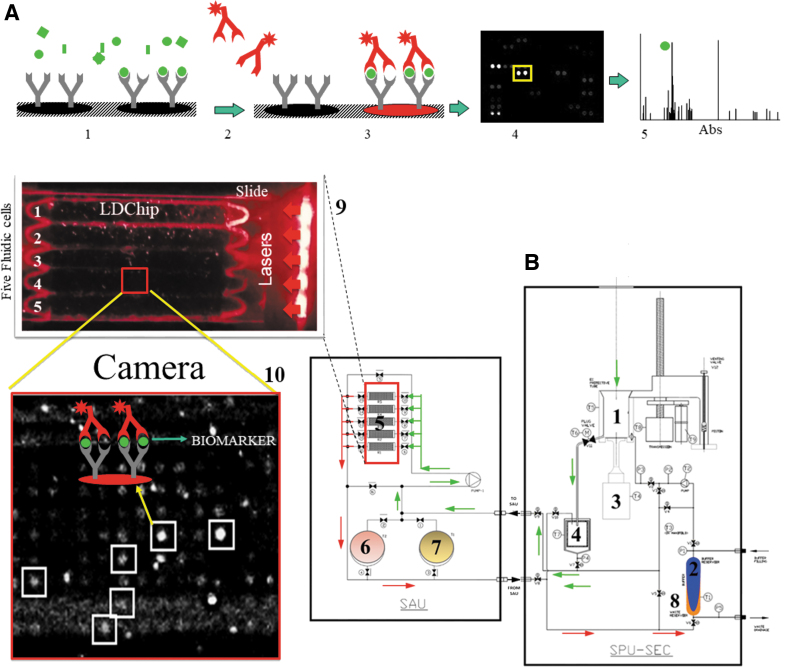
SOLID3.1 remote operation and functional diagram. **(A)** Schematic process of the FSMI: (1) A multianalyte liquid extract is incubated with the antibody microarray (the LDChip); (2) capturing antibodies on the microarray specifically bind the target molecules and positive detections are developed with a fluorescently labeled mixture of the same printed antibodies (now acting as secondary detecting antibodies) that bind the target molecules (epitope) by different positions; (3) After washing out the fluorescent antibody solution, fluorescence only remains in those capturing antibody spots that bound molecules; (4) A laser beam excites the fluorochromes and a CMOS-based camera takes a pictures that shows fluorescent spots; (5) The image is analyzed, and the fluorescence intensity quantified and plotted. **(B)** Functional diagram of SOLID instrument. SOLID consists of two units: The SPU (right) and the SAU (left). (1) EC, receives the sample from the robotic arm; (2) buffer reservoir (blue) pumped to the EC; (3) sonicator; (4) 10 μm filter; (5) recirculation chamber microarray with five flow cells; (6) buffer supply reservoir; (7) labeled antibody reservoir; (8) waste (orange) reservoir separated by a membrane from fresh buffer reservoir. The signal readout module consisting of eight diode lasers coupled with two glass cylinders illuminate both ends of the slide exciting the labeled antibodies (9); and the fluorescent signal emitted by the fluorochromes is captured by a CMOS-based camera (10). Arrows represent the direction of the flowing liquid through the flow cells (green) and direction of the residual liquid to the waste reservoir (red). EC, extraction cell; FSMI, fluorescence sandwich microarray immunoassay; SAU, sample analysis unit; SPU, sample preparation unit.

The current prototype (SOLID3.1) uses a single, reusable solvent extraction cell (EC) called the sample preparation unit (SPU) that obtains a liquid extract from a 2–3 cm^3^ sample and transfers it to the sample analysis unit (SAU), which performs immunoassays on the liquid extract (Fig. 2B). SOLID3.1 is capable of autonomously preparing and analyzing up to 5 samples obtained by a robotic drill.

The LDChip immunosensor (Parro *et al.*, 2008, 2011a; Rivas *et al.*, 2008) is the sensing core of the SOLID instrument and contains 200 polyclonal antibodies that are able to recognize a wide range of organic/biological molecules, including amino acids and peptides, proteins, exopolymeric substances (EPS), spores, and whole cells from the Bacterial and Archaeal domains (Rivas *et al.*, 2008; Sánchez-García *et al*., 2019) with limits of detection (LODs) in the ppb to ppm range (see examples in Table 1).

**Table 1.** Limits of Detection of Life Detector Chip Antibodies and Type of Immunoassay

Goal	Objectives	Targets	Example of compounds detected with LDChip	LDChip LODs	Type of immunoassay	Information obtained	References
Searching for molecular evidences of life on Mars	Contribute to the organic inventory on Mars	Complex non-volatile organic matter such as simple aromatics and PAHs	(a) Mellitic acid 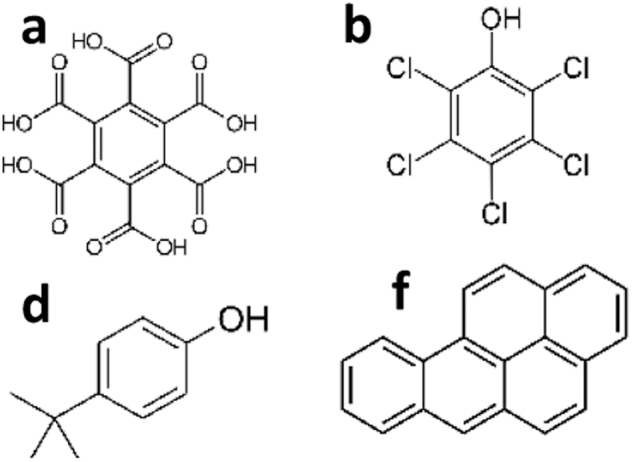	5 ppb	IMI/CMI	Oxidized organic matter	Blanco et al. (2013)
			(b) Pentachlorophenol	3 ppb	IMI/CMI		Moreno-Paz et al. (2018)
			(c) Phenylphenol	10 ppb	IMI/CMI	Relatively stable complex organic matter, regardless its origin	Fernández-Calvo et al. (2006)
			(d) 4-Terbutylphenol	100–500 ppb	IMI/CMI		Fernández-Calvo et al. (2006)
			(e) 2-Amino-naphthalene	100–500 ppb	IMI/CMI		Fernández-Calvo et al. (2006)
			(f) Benzo[a]pyrene	0.001 ppb	IMI/CMI		Moreno-Paz et al. (2018)
		Synthetic compounds containing heterocycles such as azines (C-N) or sulfur rings	(g) Atrazine 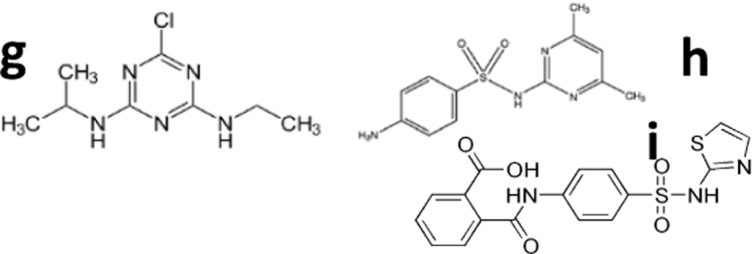	0.2 ppt to 10 ppb	IMI/CMI	Possibly biologically diagenized heterocyclic compounds	Moreno-Paz et al. (2018)
			(h) Sulfamethazine	10 ppb	IMI/CMI		Moreno-Paz et al. (2018)
			(i) Phthalylsulfathiazole	10 ppb	IMI/CMI		Moreno-Paz et al. (2018)
	Detecting molecular biomarkers	Building blocks	(j) D/L aromatic amino acids 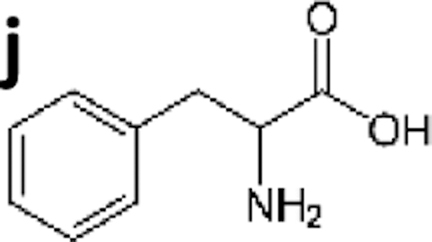	0.1–0.5 ppm	IMI/CMI	Mostly biological monomers	Moreno-Paz et al. (2018)
			(k) Thymine dimers	<5 ppb	IMI/CMI		Unpublished
		Biochemicals, biopolymers, cells, and other biochemical structures	(l) Triterpene-like (*e.g*., Finasteride) 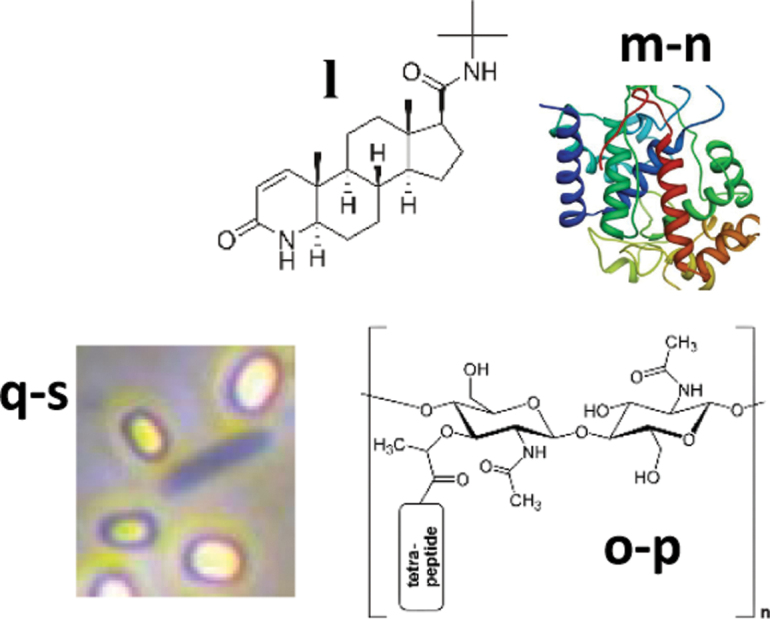	0.001 ppb	IMI/CMI	Well-preserved biochemistry from extant or recently extinct or well-preserved life remains	Moreno-Paz et al. (2018)
			(m) Peptides (5–20 amino acids)	0.1–2 ppb	FSMI/IMI		Fernández-Calvo et al. (2006)
			(n) Proteins (>20 amino acids)	0.2–2 ppb	FSMI		Moreno-Paz et al. (2018)
			(o) EPS	<10 ppb	FSMI		Fernández-Calvo et al. (2006)
			(p) Peptidoglycan	<10 ppb	FSMI		Rivas et al. (2008)
			(q) Spores and cells	10^3^–10^4^ spores/mL	FSMI		Rivas et al. (2008)
			(r) Phages	1–10 ppb	FSMI		Unpublished
			(s) Biofilms remains	1–10 ppb	FSMI		Parro et al. (2011b)

EPS = exopolymeric substances; FSMI = fluorescence sandwich microarray immunoassay; IMI/CMI = inhibition microarray inmunoassay/competitive microarray immunoassay; LDChip = LifeDetector Chip; LOD = limit of detection; PAH = polycyclic aromatic hydrocarbon.

The SPU has been designed to homogenize the sample, lyse cells, and extract organic matter into an aqueous solvent by ultrasonication and subsequent filtration to remove minerals and coarse material. The EC (No. 1 on Fig. 2B) receives from the robotic arm 0.1–1.6 g of sample and adds 5 × volume of TBSTRR extraction buffer (0.4 *M* Tris-HCl pH 8, 0.3 *M* NaCl, 0.1% Tween 20) (No. 2).

A piston moves down to seal the EC, while the ultrasonicator (40 kHz) (No. 3) makes contact with the sample through a flexible Teflon membrane. After applying several cycles of ultrasonication (300 s per cycle with a 42% amplitude), the sample is pushed forward with the piston to enter a 10 μm cylindrical filter (No. 4) to remove any coarse particulates. Both the SPU and SAU have pumps and a set of valves that allow the sample to reach the appropriate position/location and enable recirculation through different circuits.

The liquid extract is pumped into one of five microarray flow cells (No. 5) in the SAU, where organic compounds can interact with capturing antibodies printed on the LDChip microarray. The SAU has an internal recirculation circuit that enables the filtrate to re-circulate for up to 1 h to enhance the reaction kinetics between target analytes and capturing antibodies printed on the microarray. After 1 h of incubation, a liquid buffered solution (No. 6) removes any sample excess, and a mixture of fluorescent antibodies (No. 7) is pumped into the flow cell for an additional 1-h incubation to complete a sandwich microarray immunoassay.

After a final wash step with TBSTRR to remove unbound fluorescent antibodies (No. 6), the washing solution is pumped to a residual reservoir (No. 8), and a set of red diode lasers (635 nm, 180 mW; see details in Fig. 2B) illuminates the entire chip support (No. 9). The fluorescent signal emitted by the fluorochromes is captured by a CMOS-based camera that takes a picture as a tagged image file format (TIFF 16-bit; No. 10). This image is the SOLID3.1 output data that are sent to the remote science and operations team for fluorescence quantification and identification of targeted molecules by means of the GenePix Pro 7.0 software (Molecular Devices, Sunnyvale, CA) as described elsewhere (Parro *et al*., 2011a).

Before each sample analysis, a blank assay is run with only TBSTRR buffer in one LDChip flow cells following the procedure described earlier. The resultant image is used as a negative control to subtract the fluorescent spot intensities with the corresponding image obtained in the tested samples. Fluorescence intensity signals are considered positive when their value is 15% greater than the value of the mean background.

### 2.3. Field laboratory measurements to ground truth autonomous SOLID3.1 data

Replicate samples of those analyzed by SOLID3.1 were manually analyzed in the field lab by FSMI for ground truthing. In addition, sample fractions taken every 10 cm from drillholes H1 to H4 were analyzed to determine the presence of biomarkers in the study area as follows: Soil aliquots of 0.5 g were suspended in duplicate in 2 mL of TBSTRR buffer, homogenized with a hand-held ultrasonic homogenizer UP200Ht (Hielscher Ultrasound Technology) to extract the organic matter, and subsequently centrifuged at 1000 rpm for 2 min.

A total of 50 μL aliquots of each extract were injected into one of the chambers in a MAAM (which contains nine analysis chambers) and were incubated for 1 h at room temperature (Blanco *et al*., 2017b). After two washing steps with the TBSTRR buffer, 50 μL of the mixture of 200 Alexa-647 labeled antibodies were incubated with the extract in the chamber for 1 h at room temperature, washed with TBSTRR, and then scanned for fluorescence at 635 nm excitation in a GenePix 4100A scanner.

The obtained images were analyzed and quantified with the GenePix Pro 7.0 Software (Molecular Devices), and the fluorescence intensity (F) of each positive antigen-antibody pair was calculated (Parro *et al*., 2011b; Rivas *et al*., 2011; Blanco *et al.*, 2012). To eliminate false-positive signals, we considered and selected the fluorescent signal intensities for each antibody and antigen reaction as positives only when they had a fluorescence intensity signal at least 2.5 times over the background level.

### 2.4. Laboratory measurements to validate field data: protein biomarkers and metabolisms inferred from metaproteomics analysis

To assess the type and abundance of protein biomarkers that could explain many of the positive immunodetections by SOLID3.1, as well as to identify the main metabolic traits in the explored area, a metaproteomic analysis was performed on a subset of the curated samples. Curated samples from 0 to 20 cm (hole H1), 20 to 50 cm (hole H1A), and 40 to 80 cm (hole H3) were pooled into three aliquots (top, middle, and bottom samples, respectively) to obtain enough material for protein extraction and metaproteomic studies. Whole protein content was extracted by the sodium dodecyl sulfate-trichloroacetic acid (SDS-TCA) method (Giannone *et al.*, 2011; Hultman *et al*., 2015). Briefly, tubes containing equal volumes of soil and SDS buffer (4% SDS, 100 m*M* Tris-HCl pH 7.4) were submerged in boiling water for 15 min, then sonicated for 18 cycles of 30 s (40% amplitude), incubated in boiling water for another 10 min, vortexed, and centrifuged at 9000*g* (10 min, 4°C).

The supernatant was collected in a fresh tube, and extracted proteins were precipitated overnight in 20% (v/v) of TCA at −20°C and pelleted by centrifugation at 21,000*g* (40 min, 4°C, on a Beckman ultracentrifuge with a JLA-16250 rotor). Protein pellets were washed three times with ice-cold acetone, re-solubilized in 100 μL of pre-heated Molecular Biology grade water (Thermo Fisher Scientific, Inc.), and stored at −20°C. Total protein concentration was verified with Quant-iT™ Protein Assay kit (Invitrogen) according to the manufacturer's instructions and Qubit™ Fluorometer (Invitrogen). All reagents were sterilized by filtration (0.22 μm pore-sized filters) and UV exposure (5 min, *str* program, GeneLinker UV Chamber; Bio-Rad Laboratories). A blank extraction run in parallel without sample was used as a negative control for protein contamination during the extraction procedure.

The proteomic analysis was performed by liquid chromatography tandem mass spectrometry (LC-MS/MS) in the Proteomics Unit of the Complutense University of Madrid. Briefly, total proteins were first concentrated into a single band in the stacking gel of a sodium dodecyl sulfate-polyacrylamide gel electrophoresis (SDS-PAGE) gel. After cutting the protein band, proteins were eluted and digested with trypsin, and the tryptic peptides were ionized and analyzed on a Q-Exactive HF mass spectrometer (Thermo) in data-dependent acquisition (DDA) mode (Gillet *et al*., 2012). Metaproteomic sequencing chromatograms obtained with the negative control and three curated samples are shown in Supplementary Fig. S4.

The acquired MS/MS data were analyzed using Proteome Discoverer software v.2.3 (Thermo Scientific) with search engine Sequest HT to identify the peptides against SwissProt database (release May 5, 2019, 471,870 sequences), Archaea DB (February 20, 2020; 3,952,635 sequences), Fungi DB (February 14, 2020; 11,599,317 sequences), Bacteria DB (February 20, 2020; 128,912,198 sequences), and Eukaryote DB (February 20, 2020; 40,939,735 sequences) downloaded from Uniprot.org (www.uniprot.org). The acceptance criteria for proteins identification were a false discovery rate <1%, and at least one unique peptide identified with high confidence (confidence interval >95%, *p* < 0.05).

Protein spectral counts were normalized to spectral abundance factor (NSAF) as described by Zybailov *et al.* (2006). Annotation to KEGG (Kyoto Encyclopedia of Genes and Genomes) and Clusters of Orthologous Groups of proteins (COGs) databases was done on KAAS (KEGG Automatic Annotation Server) (https://www.genome.jp/kegg/kaas), using the Single Best Hit method for amino acid sequence query (Ogata *et al*., 1999; Moriya *et al*., 2007) and on EggNOG-mapper v2 server (http://eggnog-mapper.embl.de, using default settings) (Tatusov *et al*., 2003; Huerta-Cepas *et al.*, 2017).

Taxonomy, protein names, and sequences were obtained from the UniProt server (www.uniprot.com) using the UniProt Retrieve ID/Mapping tool (Bateman, 2019). To exclude any contamination introduced during sample collection or handling, proteins identified in the negative control as contamination, as well as proteins belonging to Hominidae, were subtracted from the analysis.

The sampling site also contained proteins assigned to animals, mainly Canidae, that were also eliminated. An additional filtrate was applied to eliminate proteins from common pathogens, namely Enterobacterales, Pasteurellales, Neisseriales, *Borrelia*, *Bordatella*, *Chlamydia*, *Staphylococcus*, and *Wolbachia*. Only six proteins were annotated to *Pseudomonas* in the control against 52 proteins identified in the samples. Therefore, our main analysis was performed considering *Pseudomonas*, given that they were attributed to the sample and not introduced during laboratory handling.

Venn diagrams were constructed using *BioVenn* web application (https://www.biovenn.nl) (Hulsen *et al*., 2008). The mass spectrometry proteomics data have been deposited to the ProteomeXchange Consortium via the PRIDE (Pérez-Riverol *et al.*, 2019) partner repository with the dataset identifiers PXD027518 and 10.6019/PXD027518.

### 2.5. Laboratory measurements to validate field data: microbial community composition and diversity at the study site

Total DNA was extracted for microbial community composition based on 16S ribosomal RNA (rRNA) amplicon sequence variants (ASVs) from Archaea and Bacteria. Only sediments corresponding to the samples analyzed autonomously by SOLID3.1 were investigated: from hole H1 at 10–20 cm (S-H1b), hole H1A at 40–50 cm (S-H1Ae), hole H2 at 10–20 cm (S-H2b), hole H3 at 30–40 cm (S-H3d) corresponding to playa sediments (SR), and hole H4 at 10–20 cm (S-H4b) corresponding to the alluvial fan (DF) (Fig. 1B, C).

Total DNA was extracted from 0.25 g of soil in duplicate with the DNeasy PowerBiofilm Kit (MOBIO Laboratories, Carlsbad, CA, USA) following the manufacturer's instructions. To eliminate indigenous DNA contamination from handling and buffers, two extractions were simultaneously performed with water and reagents provided in the kit and used as negative controls.

Total DNA was quantified by Qubit dsDNA HS Assay Kit (Life Technologies, USA), and then both replicates were combined (Fernández-Martínez *et al*., 2019; Warren-Rhodes *et al*., 2019) for further Paired-End Sequencing to analyze the 16S rRNA gene, via the Illumina Miseq platform (performed at the Unidad Genómica del Parque científico de Madrid).

Primers used to amplify the bacterial 16S rRNA V3–V4 gene region were 16SV3V4-Fw (CCTACGGGNGGCWGCAG) and 16SV3V4-Rv (GACTACHVGGGTATCTAATCC). Primers used for Archeal 16S rRNA gene polymerase chain reaction (PCR) amplification were Arch1F (CGGRAAACTGGGGATAAT) and Arch1R (TRTTACCGCGGCGGCTGBCA). Finally, the Eukaryotic 18S rRNA gene was amplified by using 563F (GCCAGCAVCYGCGGTAAY) and 1132R (CCGTCAATTHCTTYAART) primers.

Sequencing analyses of raw data were mainly performed with the R language for statistical computing (v.4.0.3; R Core Team, 2020). The “DADA2” R package (Callahan *et al*., 2016) was employed to group the bacterial and archaeal reads into ASVs using a custom script based upon the DADA2 Pipeline Tutorial (1.16, https://benjjneb.github.io/dada2/tutorial.html), with slight variations according to the sequencing results. Briefly, to remove low-quality base calls, reads with a number of expected errors (“maxEE” parameter) higher than 3 and 4 were trimmed from the forward and reverse reads, respectively. Taxonomic affinities were assigned by comparison of ASVs representative sequences using the ARB-Silva reference database (release 138).

In addition, a base length equal to each primer was also trimmed from the forward and reverse reads, respectively, to improve the chimera detection and removal step (“removeBimeraDenovo” command). Contaminant sequences were further identified using “decontam” R package (Davis *et al*., 2018) by employing the frequency and prevalence-based identification methods, employing two sequenced negative controls mentioned earlier (for each pair of primers) and a threshold of 0.5.

Detected contaminant sequences were removed from the dataset before subsequent analysis. After removing contaminant sequences from controls, we still found a few ASVs classified as common pathogens that were still present, probably coming from allochthonous contamination of the environment, mainly due to the presence of animals in the area or wind transport (Azua-Bustos *et al*., 2019).

Therefore, we added a new filter to remove them as we considered that they do not take part in the ecosystem (Salter *et al*., 2014; Weyrich *et al*., 2019; Zinter *et al*., 2019). As a result, we eliminated 54 ASVs (from, *i.e.*, *Corynebacterium*, S*taphylococcus*, or S*treptococcus*), and a heatmap was further created by using Statistical Analysis of Metagenomic Profiles (STAMP) software (Parks *et al*., 2014). Richness (number of ASVs), Shannon diversity (*H*′), and Evenness indices were calculated for the bacterial community with the “vegan” R package (Oksanen, 2019). Raw sequence data were deposited at the NCBI Sequence Read Archive (SRA), under BioProject ID PRJNA748352.

### 2.6. Bulk organic matter content, ion chromatography, and mineralogy

Drill samples returned to the Centro de Astrobiología laboratory in Madrid (Spain) were analyzed using diverse geochemical tools to provide a context for the field-analyzed samples. Stable isotopes of organic carbon (δ^13^C) and total nitrogen (TN; δ^15^N) were determined by isotope-ratio mass spectrometry (IRMS) following USGS (United States Geological Survey) methods (Révész *et al*., 2012) only in those samples autonomously analyzed with instruments, except for the sample S-H3h (70–80 cm), which was depleted after delivery to SOLID.

In this case, an alternative sample from hole H3 that corresponds to the middle section (S-H3d at 30–40 cm interval) was selected for the isotopic analysis. In all cases, sample duplicates of 0.5 g were homogenized by grinding with a corundum mortar and pestle. HCl was added to the samples to remove carbonates, and the samples were left to equilibrate for 24 h and then adjusted to neutral pH with ultrapure water. The residue was then dried in an oven (50°C) for 72 h or until a constant weight was achieved, and then it was analyzed in the IRMS (MAT 253; Thermo Fisher Scientific, Inc.).

The δ^13^C and δ^15^N values were reported in the standard per mil notation using three certified standards (USGS41, IAEA-600, and USGS40) and an analytical precision of 0.1‰. Total organic carbon (TOC wt %) and total nitrogen (TN wt %) were measured with an elemental analyzer (HT Flash; Thermo Fisher Scientific, Inc.) during the stable isotope measurements.

Inorganic anions and low-molecular-weight organic anions were determined by ion-chromatography. Briefly, sample duplicates (2 g) were suspended in 20 mL of deionized water for ion-chromatography (Sigma-Aldrich, Inc.) and incubated overnight in a FinePCR HAG/110V rotator (Progen). The sample was then filtered (0.22 μm PTFE filter), and the filtrate diluted 1:10 (v/v) in deionized water or analyzed directly by loading into a Metrohm 861 Advanced compact ion chromatograph (Metrohm AG, Herisau, Switzerland) by an automatic loader.

Chromatographic separation was performed in a Metrosep A sup 7-250 column (Metrohm AG) using 3.6 m*M* sodium carbonate (NaCO_3_) as eluent at a flow rate of 0.7 mL/min. All samples were analyzed in duplicate, and differences in all analytical results were always ≤5%. Their average values were used in the following sections.

Bulk minerals were identified by powder X-ray diffraction (XRD) with a Bruker D8 Eco Advance diffractometer with Cu Kα_1_ radiation (*λ* = 1.5406 Å) and a Lynxeye XE-T linear detector. The X-ray generator was set to an acceleration voltage of 40 kV and a filament emission of 25 mA. Samples were scanned between 5° (2*θ*) and 60° (2*θ*) using a step size of 0.05° (2*θ*) and a count time of 1 s, implementing the Bragg–Brentano geometry.

The phase identification was performed by comparing measured diffraction patterns (diffractograms) with X-ray powder-diffraction patterns in the PDF database with the DIFFRAC.EVA software (Bruker AXS), and a semi-quantitative calculation of their relative abundances (weight percentages) was conducted.

### 2.7. Statistical analysis

Linear regression analysis and principal components analysis (PCA) were applied to the distribution of microbial groups and metabolic activities among holes and at different depths. The calculations were run in MATLAB software.

## 3. Results

### 3.1. Autonomous SOLID3.1 detected microbial markers in the shallow Atacama subsurface

**FIG. 3.**
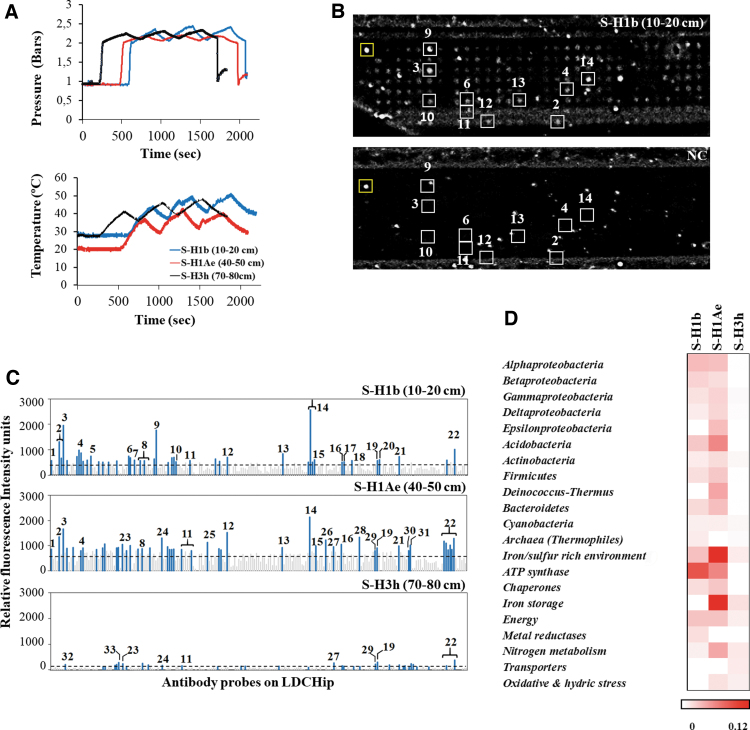
*In situ* autonomous and remote sample analysis with SOLID-LDChip. Samples (∼0.3–0.6 g) from holes H1 and H3 (S-H1b, S-H1Ae, S-H3h) were collected at 10–20, 40–50, and 70–80 cm depth respectively. **(A)** SOLID performance was checked in real time by recording the temperature and pressure inside the EC along the three cycles of ultrasonication. **(B)** Fluorescence images of LDChip after FSMI analysis of sample S-H1b (left) and the correspondent NC run just with buffer instead of sample (right). Some of the spots showing positive immunodetections are framed by white squares, whereas the yellow ones indicate fluorescent spots used as controls to frame the microarray. For simplicity, images represent just a replicate out of three in each LDChip microarray. Images were processed with a smoothing filter using ImageJ software, and fluorescence was quantified and plotted. **(C)** Immunograms from S-H1b (top), S-H1Ae (middle), and S-H3h (bottom) obtained after quantifying their corresponding fluorescence image. Positive immunodetections corresponded to antibodies raised against crude cells extracts from *Acidiphilium* spp. (1), *Magnetospirillum bellicus* strain VDYand *Magnetospirillum* spp. (2), *Polaromonas* sp. (3), *Dechloromonas agitata* strain CKB (4), *Acidithiobacillus ferrooxidans* (5), *Azotobacter vinelandii* (6), *Desulfotalea psychrophila* (7), *Geobacter sulforeducens* (8), *Frondihabitans* sp. (9), S*ulfobacillus acidophilus* (10), *Bacillus subtilis* (11), *Salinibacter ruber* PR2 (12), *Phormidium* sp. UAM361 (13), Cell extracts of Iron/Sulfur biofilms and sediments (22), *Pseudomonas putida* (23), *Streptomyces diastaticus* spores (24), *Thermus scotoductus* (25), *Burkholderia fungorum* (32), and *Colwellia psychrerythraea* (33). Antibodies raised against proteins gave positive signals for ATP synthase subunits A and B (ASF1 and ASB, respectively) (14), chaperone heat shock protein (HscA) (15), NADH dehydrogenase I (NADH) (16), Rubisco large subunit (RbcL) (17), iron reductase (FeReTs) (18); nitrate reductase subunit Alpha (NRA) (19), perchlorate reductase (295) (20), methyl CoM reductase I, beta subunit (McrB) (21), ferrodoxin (Fdx2) (26), ferritin (PfuFer) (27), nitrogen metabolism regulator (GlnB) (28), nitrite oxidoreductase beta subunit (NOR1) (29), cytochrome oxidoreductase (CydA) (30), and fructose-bisphosphate aldolase (DhnA) (31). **(D)** Heatmap of positive immunosignals detected for the main phylogenetic and functional groups as the average signal intensity of three replicates of each printed antibody. The bar represents normalized relative fluorescence intensity in a gradual color scale from white (absence of signals) to red (maximum (intensity). NC, negative control.

A total of five samples from several depths were analyzed *in situ* by SOLID3.1 aboard the K-REX2 rover (Fig. 1A–C). The instrument was remotely controlled from a nearby field station to process and assay samples (Fig. 2A, B) with the LDChip, and the corresponding fluorescent images (Fig. 3B) were uploaded daily to the mission data archive to be analyzed by the remote science team in Spain. Telemetry data show that SOLID3.1 operated optimally, as indicated by the consistent temperature and pressure values in the EC (2–2.5 bar and 30–50°C, respectively) at the end of the three sonication cycles (Fig. 3A).

LDChip targets biopolymeric microbial markers by a multiplex fluorescent immunoassay with a collection of 200 antibodies listed in the work of Sánchez-García *et al.* (2019). Positive fluorescence signals (positive immunodetections) with several antibodies were obtained in samples S-H1b (10–20 cm) and S-H1Ae (40–50 cm), with a higher signal intensity in the lower sample (Fig. 3C, D). Weak or no signals were found in samples S-H2b (10–20 cm), S-H3h (70–80 cm), and S-H4b (10–20 cm). Positive immunodetections corresponded to antibodies produced against microbial strains from the most common phyla reported in arid soils and commonly found in the Atacama Desert (Gómez-Silva *et al*., 2008; Fernández-Martínez *et al*., 2019; Warren-Rhodes *et al*., 2019; Shen *et al*., 2021), with a similar pattern in both upper samples but with higher fluorescence intensity in the middle one (40–50 cm). Similarly, some differences were observed with depth in the signal patterns from protein biomarkers, with a wider range and higher signal intensity for proteins at 40–50 cm depth (Fig. 3C, D).

Biomarkers for Actinobacteria, Proteobacteria (mainly Alpha and Betaproteobacteria classes), Firmicutes, and Bacteroidetes were relatively more abundant, whereas markers for members of the Cyanobacteria phylum (*Phormidium* spp.), as well as Gamma and Deltaproteobacteria classes, showed less fluorescence intensity (Fig. 3C, D). Markers for the diverse bacterial phylum Bacteroidetes (*Sphingobacterium* and *Salinibacter* genera) and Firmicutes (main bacteria from the *Bacillus* and *Planococcus* genera) were detected in both samples, whereas markers for the sulfide oxidizing bacteria *Sulfobacillus* sp. were only found in the upper one. Markers for *Streptomyces* spp. (Actinobacteria) spores were only present at S-H1Ae (40–50 cm).

Among Betaproteobacteria, those of the order Rhodocyclales (mainly perchlorate-reducing bacteria) were dominant at S-H1b (10–20 cm) but absent in deeper samples. In addition, immunosignals from two Deltaproteobacteria (the iron-oxidizing bacteria *Geobacter* spp. and the sulfur reducing bacteria *Desulfotalea* spp.), together with antibodies raised against a mixture of cells and exopolymeric material from an iron/sulfur rich environment, were particularly strong in S-H1Ae (40–50 cm).

**FIG. 4.**
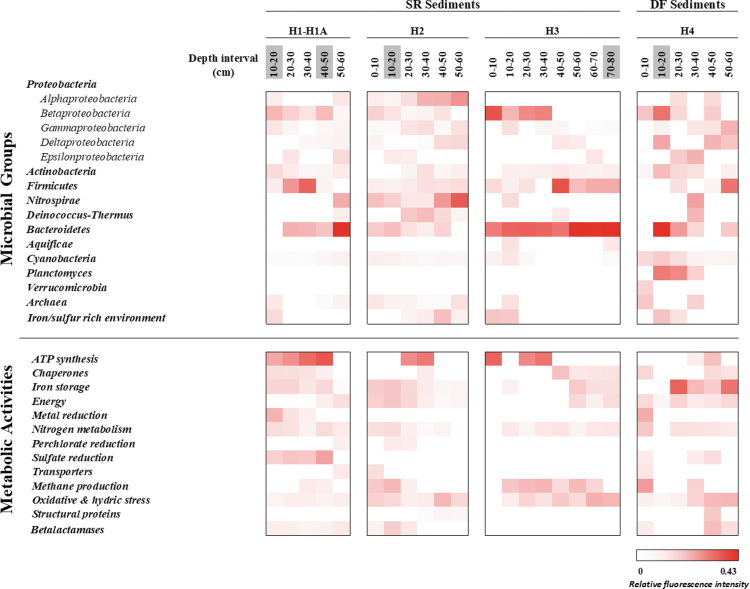
Heatmap representing the results of ground-truth analyses based on the LDChip immunoassay. The antibodies were classified in different microbial groups among holes and depths on the basis of the phylogeny of the target used for producing each antibody in the LDChip200 (more information as described in Sánchez-García et al., 2018). Similarly, proteins detected as positive immunosignals were grouped in metabolic activities based on their molecular functions or different biological processes. Positive antigen-antibody reactions in SR and DF sediments were expressed as the average signal intensity of six replicates of each printed antibody. The bar represents normalized fluorescence intensities in a gradual color scale from white to red, where white color indicates the absence of signals and red color the intensity of positive signals up to a maximum (0.43 of Relative fluorescence intensity). Depth intervals highlighted in gray correspond to samples that were delivered and analyzed with SOLID3.1 instrument.

LDChip also detected the presence of proteins or their remains in S-H1b (10–20 cm) and S-H1Ae (40–50 cm) samples, with similar patterns for proteins related to ATP synthesis (ATP synthase and NADH dehydrogenase I), iron reductase (FeReTs), nitrate reductase (NRA), methyl CoM reductase I (McrB), and chaperones (HscA). Some proteins were only detected in S-H1b, such as Rubisco protein (RbcL) and perchlorate reductase (295), whereas a ferredoxin (Fdx2), a nitrogen regulatory protein P-II (GlnB), and nitrite reduction (nitrite oxidoreductase, NOR1) were only detected in S-H1Ae (Fig. 3C, D). Although the LDChip immunosignals autonomously detected with SOLID3.1 showed low intensity, these results agreed with those obtained by ground-truth analysis carried out in parallel at the field lab (Fig. 4).

### 3.2. Field laboratory measurements to ground truth autonomous SOLID3.1 data

Samples from all drillholes were collected every 10 cm and immediately brought to a field lab for a thorough immunoassay study for ground truth of SOLID3.1 results. Antibodies and positive immunodetections were classified in two groups, following the immunogens used to produce them. The first group (“microbial groups”) consisted of microbial strains of different phyla of bacteria and archaea based on the NCBI database (www.ncbi.nlm.nih.gov/taxonomy; December, 2019) and the second group (“metabolic activities”) consisted of the main metabolisms or biological processes associated with the detected proteins. A heatmap shows the positive immunodetections for the main microbial groups (Fig. 4).

Ground-truth analyses corroborated and expanded the autonomous SOLID3.1 results with more accurate and new immunodetections. Field lab immunograms yielded the strongest signals at all depth intervals in drillhole H2. Drillholes H1A and H3 yielded weaker, though comparable, signal patterns. Biomarkers from Proteobacteria (mainly Betaproteobacteria class), Firmicutes, and Bacteroidetes were dominant in all drillholes.

Betaproteobacteria markers dominated the first 40 cm, whereas markers for Deltaproteobacteria and Bacteroidetes yielded the strongest signals from 40 to 60 cm. Conversely, fewer intensity signals were detected for Actinobacteria markers in all drillholes, with similar fluorescence intensity values at all depths. Unlike the rest of the holes, H2 showed other positive immunosignals that indicate the presence of Nitrospira and Deinococcus-Thermus phyla as well as the class Alphaproteobacteria. Archaea markers were detected at all depths only in drillhole H2.

The higher differences in the microbial biomarker composition were found in drillhole H4, located outside the evaporitic playa, on the alluvial fan (Fig. 1B, C). Samples from H4 did not show any discernible immuno-patterns with depth, showing a random distribution of microbial biomarkers except for Cyanobacteria, which showed higher fluorescence signals in the top 30 cm (Fig. 4) and Planctomyces markers, which were detected between 10 and 40 cm depth samples. Ground-truth analysis results showed a similar pattern in protein immunoprofiles between SR and DF sediments, with higher similarities between drillholes H1, H2, and H3 compared with the regolith-like drillhole H4 (Fig. 4 and Supplementary Tables S1 and S2 and Supplementary Fig. S1), although these differences were less remarkable than those found for the phylogenetic profile.

Markers for nitrogen metabolism, energy production, iron storage, and oxidative stress were inferred in all samples. A higher protein abundance was observed in the SR sediments, with the most predominant proteins at all depths related to nitrogen metabolism and oxidative stress. Markers for nitrogen fixation (NifD, NifH, NifS, and GlnB proteins) were dominant in the upper part of the holes. However, markers for nitrate reduction (mainly NirS and NOR1 reductases) were present from 50 to 80 cm.

Proteins involved in biological processes related to the synthesis/hydrolysis of ATP such as Asf1 and ASB (alpha and beta subunits of ATP synthase, respectively) gave stronger signals up to 40 cm depth, whereas those involved in oxidative stress, such as an oxidoreductase (CydA), a polyhydroxybutyrate (PHB) synthetase subunit (PhaC1), a superoxide dismutase (SodA), and an iron stress-induced chlorophyll-binding protein (IsiA1), dominated in deeper samples (30–80 cm). In addition, positive immunodetections related to sulfate reduction (DsrB protein) were particularly strong in drillhole H1, together with a methane production protein (McrB protein) that was detected in all drillholes.

The proteomic immunoprofile shows a slightly different pattern in drillhole H4, where only protein markers related to iron storage (ferredoxin), energy (Rubisco and isocitrate dehydrogenase), and nitrogen fixation were broadly detected, whereas markers for oxidative stress proteins (CydA and PhaC1) were only present at 40 cm depth.

From data obtained from the mineralogical and geochemical analyses (see results section 3.5) and the MAAM and LDChip immunoprofiles (Fig. 4), we found significant homogeneity between drillholes H1-H1A, H2, and H3 (SR sediments), particularly in terms of the high concentration of sulfates in comparison to the basement desert soil (DF sediments). To verify this grouping, we calculated the linear correlation *r* and its associated Pearson's correlation coefficient between drillholes at depths from 10 to 60 cm (Supplementary Tables S1 and S2) based on the field lab immunoprofiles.

This calculation shows a strong cross-correlation between drillholes H1-H1A, H2, and H3 as being especially relevant between drillhole H3 and the rest (*p*-value_H1–H3_ = 2 · 10^−8^ and *p*-value_H2–H3_ = 7 · 10^−4^). In contrast, drillhole H4 showed no correlation with drillholes H1-H1A and H2 (*p* = 0.18 and *p* = 0.68, respectively), whereas some correlation was observed between drillholes H3 and H4 (*p* = 0.001).

Further, we conducted a PCA to compare how depth affects the population diversity between the four drillholes H1-H1A, H2, H3, and H4 (Supplementary Fig. S1). The results show a similar first principal-component pattern between drillholes H1-H1A and H3, reflecting a strong positive correlation between the populations at all depths in both holes.

In drillhole H2, very similar first and second principal-components patterns were found between the samples at 20–30 and 30–40 cm, and between 40–50 and 50–60 cm, these two similarities could also be clearly detected in H3. The erratic immunoprofile patterns in drillhole H4 (where we have to focus on the third principal component to account for a significant fraction of the variance) were drastically different from those of the rest of the holes.

Taking all this into account, we combined and considered all samples from SR sediments as replicates of a single, unique Playa “basin” sample, while the remaining group of samples (*i.e.*, DF) is only represented by H4. Thereby, samples processed and analyzed with the instruments integrated into the K-REX2 rover could also be correlated with samples returned to and analyzed by DNA sequencing and metaproteomics in the laboratory.

### 3.3. Laboratory measurements to validate field data: protein biomarkers and metabolisms inferred from metaproteomics analysis

We obtained 1.45, 0.85, and 0.87 μg/g of total protein from top, middle, and bottom pools of samples (see Section 2.5), respectively. After the metaproteomic process to eliminate proteins present in the control sample (Supplementary Fig. S2), the taxonomic composition of samples without contaminants calculated on NSAF showed that Eukarya followed by Bacteria dominated the SR sediments (Supplementary Fig. S3).

A total of 182 proteins were identified and assigned to Archaea (14.8%), Bacteria (35.7%), and Eukarya (49.5%), whereas 19 of them were unknown. Then, a new filter was applied to eliminate eukaryotic proteins (mainly assigned to the Animalia kingdom; Supplementary Fig. S4) and proteins from common pathogens (see Section 2), and a final set of 48 unique proteins was obtained (Supplementary Table S4).

**FIG. 5.**
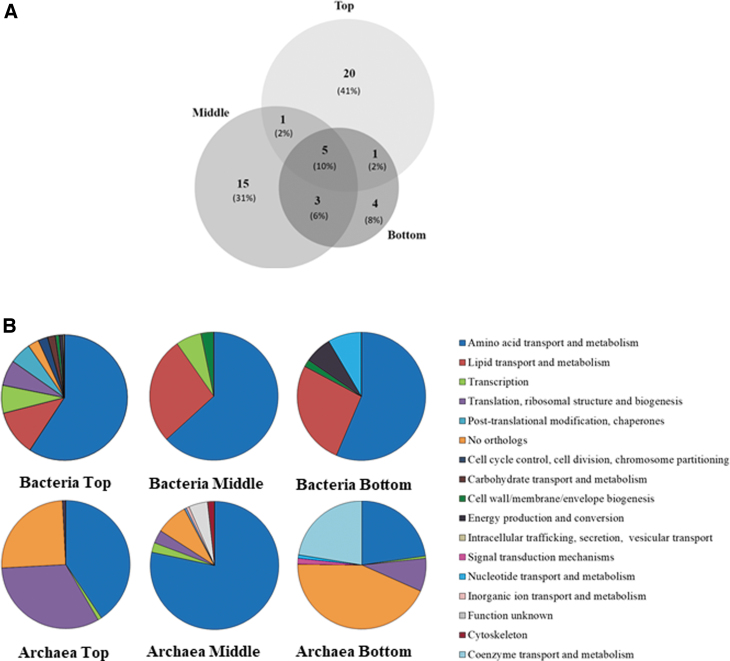
Metaproteomics revealed metabolic and biodiversity features in the study area and corroborated SOLID results. **(A)** Venn diagram representing the unique protein composition on top (10–20 cm), middle (20–50 cm), and bottom (40–80 cm) samples, based on UniProt identifiers. **(B)** Distribution of proteins among the general cellular metabolic functions as deduced from the COGs categories in top (hole H1A, 10–20 cm), middle (hole H1A, 20–50 cm), and bottom (hole H3, 40–80 cm) samples according to COG database, calculated as the percentage of NSAF after eliminating common pathogens (Enterobacterales, Pasteurellales, Neisseriales, Borrelia, Bordatella, Chlamidia, Pseudomonas, Staphilococcus, and Wolbachia). COGs, Clusters of Orthologs Groups of proteins; NSAF, normalized to spectral abundance factor.

Out of the identified proteins, 41% (20 proteins) were unique to the top pool of samples (10–20 cm), 31% (15 proteins) to the middle (20–50 cm) sample, and 8% (4 proteins) to the bottom sample (40–80 cm). Only 10% of the identified proteins (five proteins) were shared among the different depths based on the UniProt Knowledge database (Bateman, 2019) (Fig. 5A).

Despite the low number of proteins identified in the Playa (only 48 filtered entries), identified proteins assigned to the prokaryotic (bacterial and archaeal) community were annotated and categorized according to their functions by using the Clusters of Orthologous Groups of proteins (COGs) database, and classified into 17 functional categories (Fig. 5B). Proteins assigned to Bacteria mainly showed a greater diversity of functional categories in the top and middle pools, whereas the bottom sample showed the lowest diversity.

By contrast, those from Archaea showed differences in the pattern of metabolic functions inferred from the proteins in the three pools (Fig. 5B). The COG analysis revealed that the most abundant cluster in the prokaryotic community belonged to amino acid transport and metabolism. Other main metabolic categories assigned to bacteria were (1) lipid transport and metabolism, and (2) transcription, (3) cell wall, membrane, and envelope biogenesis.

Conversely, COGs assigned to Archaea were enriched in biological processes related to (1) translation, ribosomal structure, and biogenesis, and (2) coenzyme transport and metabolism, with a high proportion of sequences classified as a non-orthologous cluster in top and bottom pools (25% and 44%), respectively.

The majority of protein markers corresponded to amino acid metabolism that were homogeneously distributed in all depths. It is remarkable that the relatively high abundance of serine hydroxymethyltransferase (SHMT) assigned to Alphaproteobacteria (represented by the O. Rhizobiales) and Deltaproteobacteria (O. Desulfovibrionales) was found only in the top pool, whereas it was assigned to the O. Syntrophobacterales in the middle and bottom pools of samples (https://www.ebi.ac.uk/pride); the enzyme catalyzes the reversible conversion of L-serine to glycine and tetrahydrofolate (THF) to 5,10-methylenetetrahydrofolate. In addition, a glycine cleavage system protein H was detected in the top sample. Both proteins are important in DNA synthesis by providing substrate to one-carbon cellular pathways in growing cells. (Sodolescu *et al*., 2018). Another abundant protein assigned to Archaea was associated with histidine biosynthesis and dominated middle and top samples (78% and 41%, respectively).

Two proteins assigned to transcription functions were also detected in SR sediments. A nitrogen regulatory protein P-II (GlnB) in the top pool regulates the activity and concentration of glutamine synthetase (GS) as well as a protein identified in the three pools, and annotated to the perchlorate reducing archaeon *Aeropyrum camini*, that further validate the LDCHip results on perchlorate reduction (Liebensteiner *et al*., 2015). Only one protein, related to isoprenoid synthesis, was clustered in the lipid transport and metabolism.

The presence of pyruvate dehydrogenase, crucial in the acetyl-CoA production to be used in the citric acid cycle and involved in energy production and conversion functions, was only present at the top pool and identified as belonging to members of Actinobacteria and Halobacteria. Also, two ATP synthase (alpha subunit and V-type subunit I) proteins were only detected in the top pool as well as by SOLID and ground-truth immunoassays.

Other proteins detected corresponded to translation, ribosomal structure, and biogenesis (mainly at bottom and middle pool samples), along with another relevant set of proteins such as chaperonine, superoxide dismutase, and catalase, involved in the response to stress conditions (mainly in the upper samples).

Proteins assigned to the Archaea domain were mainly involved in amino acid transport and metabolism in the three pools of samples, but especially in the middle section, representing 78% of the total. As in Bacteria, the biosynthesis of histidine was highly represented in the top sample, as well as proteins involved in protein biosynthesis and assigned to translation, ribosomal structure, and biogenesis.

Unlike in Bacteria, the bottom sample was especially abundant in proteins not associated with any COG (44%), followed by the pyridoxal 5′-phosphate synthase subunit (PdxS), a protein related to coenzyme transport and metabolism (23%) and assigned to the archaea *Sulfolobus*. This protein is involved in the synthesis of the essential cofactor for a large number of enzymes.

**FIG. 6.**
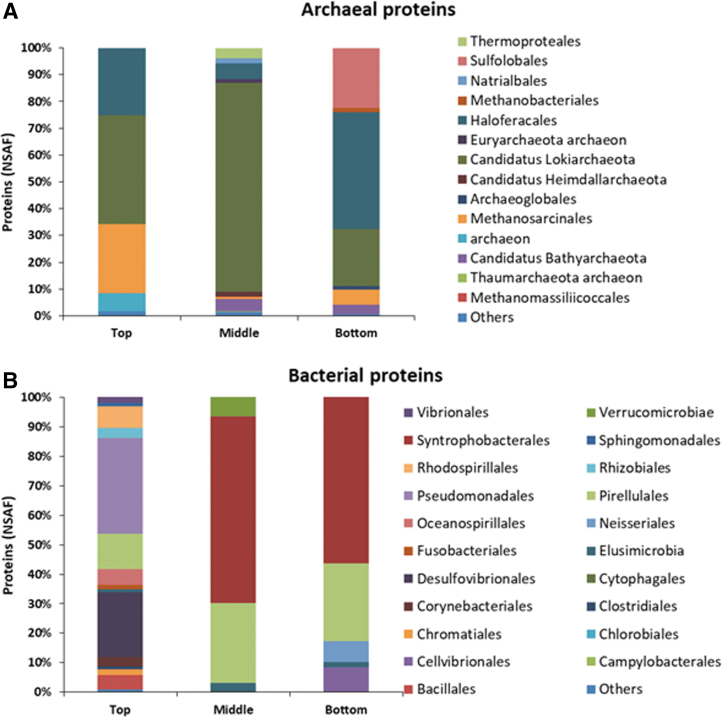
Microbial phylogenetic distribution in SR sediments based on the metaproteomic analysis. **(A)** Identified archaeal and **(B)** bacterial proteins as relative abundance of NSAF for samples top, middle, and bottom. Orders representing less than 1% were grouped under the “other” category.

Finally, the phylogenetic distribution of prokaryotic proteins showed a distinct microbial composition between the top, middle, and bottom samples (Fig. 6). Playa sediments were mostly dominated by Archaea in middle and bottom samples (94% and 79% of the total proteins, respectively). Candidatus Lokiarchaeota was the most abundant class in the three pools of samples, especially in the middle sample (78%), followed by the order Haloferacales, which represented 25% and 44% in the top and bottom samples, respectively. By contrast, Methanosarcinales were detected in the top pool (25%) whereas the order Sulfolobales (23%) was only present in the bottom pool (Fig. 6A).

Bacterial proteins were mainly assigned to Proteobacteria and Planctomycetes phyla in the three pools of samples. Shallow sediments (Top sample) showed the highest bacterial diversity, with 76% of the members assigned to the Proteobacteria phylum, mainly Gammaproteobacteria class (*Psychrobacter* [42%]), followed by Deltaproteobacteria class (Delsulfovibrionales, 22%), and Alphaproteobacteria class (Rhodospirillales and Rhizobiales were the dominant orders with 11% of the assigned peptides).

**FIG. 7.**
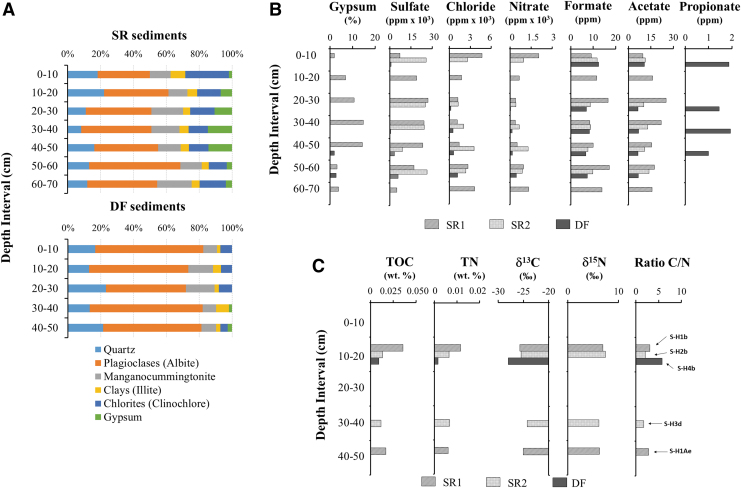
Mineralogy and bulk geochemical profile of sediments at the Playa area. **(A)** Mineralogical composition of material retrieved from the robotic drill was determined by powder XRD. Mineral composition for SR sediments represents the average values of sites SR1 and SR2. **(B)** Gypsum content (%), anions, and small organic acids concentrations in the drilling sites (SR1, SR2, and DF sediments) were measured by XRD and IC at 10 cm intervals, respectively. **(C)** TOC, TN, and isotopic fractions content were only measured in samples delivered to SOLID and other instruments. The arrows show each sample at its corresponding depth interval. All the experiments were performed in duplicate, and the error found between samples was always lower than 5%. Arrows indicate values for samples directly analyzed with SOLID instrument. IC, ion chromatography; TN, total nitrogen; TOC, total organic carbon; XRD, X-ray diffraction.

Planctomycetes represented 11.75% (Pirellulales) followed by Firmicutes, which were present only in 5.78%. By contrast, the Deltaproteobacteria class (Syntrophobacterales) was the most abundant in the middle (63%) and the bottom samples (53%) where sulfate concentrations reach higher values (Figs. 6 and 7B) followed by Planctomycetia class (Pirellulales).

Notably, Actinobacteria (10%), which was dominant by DNA sequencing and detected at all depths with the LDChip, represented only 3.7% in the top pool and was absent in the middle and bottom pools of samples by metaproteomics (Fig. 6).

### 3.4. Laboratory measurements to validate field data: corroborating the presence of microbial groups with DNA sequencing

Despite the low sample availability and the low amount of DNA retrieved from the samples collected with the rover and drilling system (300–1100 pg/g of soil), sequence data revealed the presence of the main microbial groups found with SOLID-LDChip and metaproteomics shown earlier. Bacteria was the primary domain detected in the Playa area, whereas Archaea were only detected in one of the five samples analyzed.

Conversely, the analysis of the eukaryotic community with specific primers did not show reliable results, as has also been reported (Schulze-Makuch *et al*., 2021). Taxonomy diversity in the five rover autonomous samples was evaluated using alpha diversity indexes based on ASVs, which indicated a bacterial population with low diversity and even bacterial richness (S) similar among all samples (Supplementary Table S3).

In agreement with Warren-Rhodes *et al*. (2019), small differences were found between the SR and DF sediments (sample S-H4b), the latter presenting the highest number of observed ASVs (54 ASVs) and therefore, the higher diversity (Shannon index = 2.84). The Shannon diversity index (*H*′) values showed a discernible pattern among samples in SR sediments, with similar values for the first 40 cm (1.88 and 2.13 in S-H1b and S-H3d, respectively), whereas samples S-H2b and S-H1Ae showed the lowest values (Supplementary Table S3). Evenness index indicated that the most equally bacterial distribution was present in SR sediments up to 40 cm (samples S-H1b, S-H2b, and S-H3d).

Differences in alpha diversity indexes between both SR and DF sediments were clear when comparing the primary phyla and classes present in them (Supplementary Fig. S5). DF (sample S-H4b) and SR sediments (samples S-H1b, SH2b, S-H3d, and S-H1Ae) showed a relatively similar phylum composition in the most representative groups, reflecting a low diversity and differing only in their respective proportions.

However, DF sediments (10–20 cm) showed a higher number of different phyla (*e.g.*, Chloroflexi phylum, order Kallotenuales represented 10%), whereas the SR samples were dominated by three main phyla (Actinobacteria, Proteobacteria, and Firmicutes). Actinobacteria (70% and 53% in S-H1b and S-H3d, respectively) and Proteobacteria (18–38%, up to 40 cm) were the most abundant phyla, and to a lesser extent, Firmicutes, in agreement with ground-truth data analysis (Fig. 4).

In addition, a different pattern in the bacterial classes was obtained between shallow sediments and 40 cm depth (samples S-H1b, S-H2b, S-H3d) and sample S-H1Ae (40–50 cm). Among the Proteobacteria, Alphaproteobacteria nitrogen fixers (Rhizobiales) predominated in samples S-H2b and S-H3d (up to 40 cm), while members of the Azospirillaceae family were found in a lesser proportion (sample S-H3d).

Surprisingly, nitrogen fixers were absent in S-H1b, which was dominated by *Paracoccus* sp. (Rhodobacteraceae), also detected by SOLID-LDCHip (Fig. 3). Gammaproteobacteria were detected in shallow sediments, mainly in sample S-H1b (including the DF sediments) but were absent in sample S-H1Ae (40–50 cm depth). The order Burkholderiales was found in both SR and DF sediments, whereas the order Xanthomonadales was only identified in the upper layers of SR sediments (sample S-H1b; 10–20 cm).

Unexpectedly, Beta and Deltaproteobacteria were not detected in amplicon sequencing analyses, although they were found by metaproteomics. In addition, positive immunosignals were obtained with SOLID and by ground-truth analysis for both Proteobacteria classes, and in all drillholes, the former being dominant in the upper layers and the latter only detected from 40 cm downward.

Actinobacteria was the dominant phylum in both S-H1b (69%) and S-H4b samples (77%), with members of Thermoleophilia, Rubrobacteria, Acidimicrobiia, and Actinobacteria classes in sample S-H4b, whereas sample S-H1b was dominated by members of Micrococcales (*i.e*., *g. Kocuria*) and Pseudonocardiales orders. Conversely, Actinobacteria were absent in sample S-H1Ae.

Firmicutes was less abundant in SR sediments. Firmicutes was dominated by the Bacilli class with a homogeneous distribution in samples S-H1b and S-H3d (4–9% of 16S rRNA sequences on average), being dominant in sample S-H1Ae (57%). Bacteroidetes were absent in SR sediments, although high immunosignals were found with the LDChip immunosensor at all depths, especially in hole H3 (Fig. 4).

Notably, and probably due to the small number of sequences retrieved from this sample, *Sulfurihydrogenobium* sp., a member of the phylum Aquificae, was the only ASV detected in sample S-H1Ae (Supplementary Fig. S5). Finally, although cyanobacterial markers were detected with the LDChip immunosensor in low proportion in all drillholes, no DNA sequences were found nor proteins in metaproteomic studies (Fig. 6).

### 3.5. Laboratory results to validate field data: bulk organic matter, ion chromatography, and mineralogy

The mineral composition of playa samples determined by XRD analysis (Fig. 7A) was consistent with an evaporitic deposit mainly composed of alluvial and colluvial sediments. Hydrated sulfates (*e.g.*, gypsum) were present at relative abundance >10% in SR sediments (from 10 to 50 cm depth), but their abundance was lower (<3%) in DF regolith (hole H4) and they were only detected at 40–50 and 50–60 cm depth intervals. These results are in agreement with those recently found in the Yungay region and other playas (López-Lozano *et al.*, 2012; Fernández-Martínez *et al*., 2019; Shen et al., 2021).

The analysis of soluble anions by ion chromatography showed that sulfate predominates in SR sediments, with concentrations ranging from 10 to 30 × 10^3^ ppm (mg/kg) versus 0.6 to 3.8 × 10^3^ ppm for DF regolith (Fig. 7B). Chloride and nitrate were also abundant in SR sediments, with values close to 2362 ± 833 (SR1) and 2885 ± 1245 ppm (SR2) on average, respectively.

The drillhole H4 (DF) showed the lowest concentrations of chlorite and nitrate at 10–20 cm depth (sample S-H4b) with 4.5 and 12.54 ppm respectively, which increased with depth to the highest concentrations at 40–50 cm. Unlike other playas, where phosphates were abundant at all depths, we did not detect phosphate in any sampling site (Warren-Rhodes *et al*., 2019). Similarly, analyses of small organic acids revealed the presence of formate and acetate ions at all depth intervals, with average values of 17.35 ± 4.9 ppm, 12.4 ± 3.6 ppm, and 8.3 ± 2.3 ppm for SR1 (deep basin), SR2 (shallow basin), and DF sediments, respectively. Traces of propionate were present only in DF regolith (1.3 ± 0.8 ppm) (Fig. 7B).

Organic matter analyses revealed low TOC (0.009–0.036%) and TN (0.002–0.012%) in all samples, with the highest TOC (0.036%) and TN (0.012%) observed at 10–20 cm depth (sample S-H1b) (Fig. 7C). Bulk isotopic analyses (δ^13^C) of the samples revealed similar δ^13^C values at all depths in SR sediments (Fig. 7B), with a slight enrichment of ^13^C at 10–20 cm depth in sample S-H1b (0.036%). Similarly, all SR samples also showed comparable δ^15^N values. In contrast to SR, only one DF sample at 10–20 cm depth (S-H4b) showed a relatively more depleted δ^13^C value (−28‰), whereas δ^15^N was not detected.

## 4. Discussion

### 4.1. Remote life detection and microbial biomarkers profiling with SOLID-LDChip

The findings of sulfate minerals such as gypsum and their ubiquitous presence on the surface of Mars have been associated with the presence of liquid water on Early Mars (Langevin *et al.*, 2005; Kounaves *et al.*, 2010; Vaniman *et al.*, 2014). Terrestrial playas, “dry lakes,” or “paleolake basins” are a promising target for the search for extant or extinct life on Mars, particularly due to their important preservation role (Cabrol and Grin, 1999; Farmer and Des Marais, 1999; Baldridge *et al.*, 2004; Wilhelm *et al.*, 2017) as well as for testing instrumentation developed for exploration and life detection on Mars (Sutter *et al.*, 2002; Warren-Rhodes *et al.*, 2007, 2019; Fernández-Martínez *et al.*, 2019).

Evaporitic systems, widespread in the Atacama's hyperarid core, have been investigated in several earlier analog studies (Parro *et al.*, 2011b; Davila *et al.*, 2015; Meslier *et al.*, 2018; Sánchez-García *et al.*, 2018; Wilhelm *et al.*, 2018; Fernández-Martínez *et al.*, 2019). To date, several in-field experiments have been developed in the Atacama soils for testing the robustness and autonomous operation of instruments aimed for planetary exploration missions (Cabrol *et al.*, 2007; Hock *et al.*, 2007; Glass *et al.,* 2014; Fernández-Martínez *et al.*, 2019; Warren-Rhodes *et al.*, 2019).

We have proposed the use of molecular recognition, particularly antigen-antibody interaction, as the analytical method for detecting organic molecules and life in planetary exploration (Parro *et al.*, 2005a, 2008, 2011a; Moreno-Paz *et al.*, 2018). In fact, antibodies are robust enough to withstand long-term storage, temperature cycles, and radiation exposure equivalent to missions to Mars (de Diego-Castilla *et al.*, 2011; Derveni *et al.*, 2012; Coussot *et al.*, 2019).

Over the past decade, we have developed a set of more than 200 antibodies that facilitate the recognition of well-selected organic molecules and microbial markers, which have been implemented into the LDChip and SOLID instrument for remote sample processing and analysis. Although we have previously tested SOLID2 (Parro *et al.*, 2008) and SOLID3 (Parro *et al.*, 2011a; Sánchez-García *et al.*, 2020) prototypes in the field, herein we report for the first time an end-to-end remote mission simulation with SOLID3.1.

The simulation was part of the ARADS project 2019 field drilling campaign with rover K-REX2 equipped with analytical instrumentation for geochemistry and life detection. A small evaporitic playa was explored by performing several remote drillings, sampling, and analysis as directed by a remote science team (Figs. 1 and 2). SOLID was successfully loaded and remotely operated, and it detected, in near real-time, several key molecular biomarkers at different depths (Fig. 3B, C). Though SOLID was only able to analyze three of the five samples selected for *in situ* analysis, we were able to identify critical operation requirements, including loading the right amount of sample (∼0.5 cc) such that liquid solvent volume ratio (5–6 mL/g^-^) could be appropriate for extraction. In some cases, the loading system failed and no sample entered the SOLID EC.

We are currently working on a robust dosing system that will ensure the proper amount of sample is loaded, so that overloading and clogging does not occur in the EC. In addition, we identified other steps that need to be addressed as follows: (1) The efficiency of sonication should be improved by reaching the upper limits of temperature and pressure in less time (Fig. 3A); (2) Sample filtering should be speeded up (currently 30–40 min timeframe); (3) The washing out process should be improved to remove the still-high background per spot; (4) The efficiency of fluorochrome excitation with lasers should be optimized; and (5) The image capturing system should be optimized as well to lower the unspecific background light as much as possible.

In the present study, we have shown that SOLID-LDChip addresses several features of the ladder for life detection and fulfills several of the required criteria for instrument performance as described by Neveu *et al.* (2018). The LDChip can detect complex organics from polycyclic aromatic hydrocarbons (PAHs), peptides, oligopolysaccharides, nucleic acids, proteins, or cellular remnants that inform properties of life such as structure, replication, metabolic function, or even phylogeny (Sánchez-García *et al.*, 2019; Supplementary Tables S1 and S5).

Although the LDChip targets terrestrial-like life, it can also detect non-biological organics or even extraterrestrial compounds found in meteorites with LOD at the ppb or even ppt level (Moreno-Paz *et al.*, 2018) (Table 1). In this field work, even without optimal instrument performance, SOLID-LDChip detected signs of life by showing positive immunodetection of microbial markers associated with members of Actinobacteria, Proteobacteria, Firmicutes, and Bacteroidetes phyla, as well as proteins known to be involved in certain metabolisms such as the nitrogen cycle (nitrogen fixation and nitrate reduction) or cellular energy production (Supplementary Table S5). Moreover, SOLID-LDChip provided a vertical biomarker profile that later was corroborated with a ground-truth sample analysis (in the field and in the lab), and further validated and complemented with DNA sequencing and metaproteomics.

Every technique has its own constraints, drawbacks, and bias, which could explain some of the differences found in the results obtained between them (Blanco *et al.*, 2017a). Differences among techniques include the amount of sample, the processing (lysis, extraction, or solvent) of the sample, the target of the analytical technique (DNA, proteins or antigens), or multiple steps with the inherent loss of efficiency in each one. In addition, protein identification depends on the complexity and heterogeneity of the environmental communities and the limitation to assign taxa by the species present in the databases. Although protein databases are increasing in recent years, they are still under construction, and thus, metagenomic data collected from different environments are being used as the reference. As a result, peptide sequence matches and protein coverage can be poor and can explain the dissimilarities obtained between amplicon sequencing and metaproteomics.

LDChip, as a multiplex antigen assay, is highly reliable in both sensitivity and specificity (Table 1). However, like every technique devoted to searching for life on another planet, it requires corroboration with redundant data or other instruments. Indeed, in this campaign, there was a suite of instruments and techniques for detecting life-related activities, such as capillary electrophoresis, which detected amino acids in the same samples as SOLID (Mora *et al.*, 2020), or the ATP assay used in other campaigns (Bonaccorsi and Stoker, 2008).

In a mission to Mars, LDChip could always be complemented with other techniques to corroborate the presence of organic matter (gas chromatography-mass spectrometry or Raman and infrared spectroscopy). Indeed, we propose as a minimal set of instruments in a mission for life detection: (1) a chemical laboratory for detecting anions and cations, similar to the Phoenix lander Wet Chemistry Laboratory (WCL) (Kounaves et al., 2010) to characterize the chemistry; (2) a GC-MS for detecting small size compounds such as amino acids, aromatics, aliphatic hydrocarbons, or a capillary electrophoresis for detecting amino acids and enantiomeric properties such as MILA, and; (3) SOLID-LDChip for detecting polymers such as proteins, nucleic acids, polysaccharides, cell remains, or even aromatic amino acids or other small compounds in large complexes or at the surface of nano organo-mineral particles.

With all three, we could cover three important features: simple chemistry, biochemical monomers, and finally, complex biochemistry, as proposed in the IceBreaker mission concept to Mars (McKay *et al.*, 2013).

Finally, one of the main advantages of the SOLID instrument is its versatility, that is, the separation into two physical and functional units allows the SPU to prepare samples for feeding other instruments; for example, by extracting nucleic acids to feed the MinION DNA sequencing instrument, as already reported (Maggiori *et al.*, 2020), or by preparing extracts for in-line microfluidic Raman spectrometry (Fairén *et al.*, 2020).

### 4.2. Microbial community and metabolisms in the shallow subsurface of the Atacama evaporitic playa

The core of the Atacama Desert is composed of soils with a sparse content in organic matter, and it harbors a limited microbial mass (Navarro-González *et al.*, 2003; Azua-Bustos *et al.*, 2012, 2017; Crits-Christoph *et al.*, 2013; Schulze-Makuch *et al.*, 2018; Warren-Rhodes *et al.*, 2019; Knief *et al.*, 2020; Shen *et al.*, 2021), as observed here (TOC values ≤0.036%).

Still, SOLID was able to detect *in situ* microbial molecular markers assigned to several groups of microorganisms as well as protein markers for particular metabolisms that might be operating at the time of sampling or possibly a reflection of the recent rain events in 2015 and 2017 (Wilhelm *et al.*, 2017; Azua-Bustos *et al.*, 2018; Schulze-Makuch *et al.*, 2018, 2021; Fernández-Martínez *et al.*, 2019).

To the best of our knowledge, few studies have characterized the microbial composition of shallow sediments (0–80 cm), emphasizing the presence of depth-associated microbial profiles driven by available water content and increased soluble salt concentrations (Meslier *et al.*, 2018; Fernández-Martinez *et al*., 2019; Warren-Rhodes *et al.*, 2019).

**FIG. 8.**
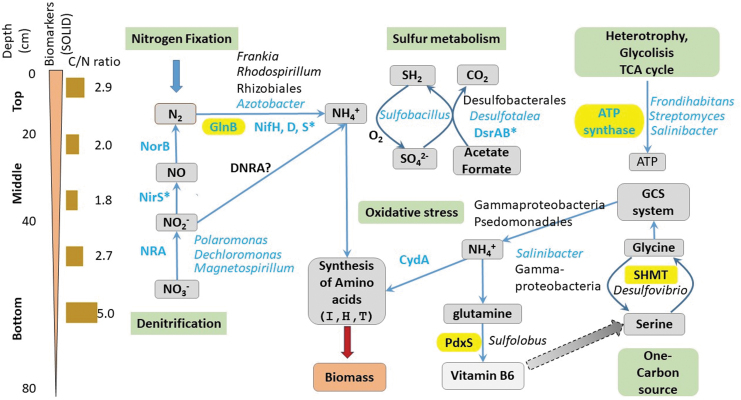
Changing microbiological and metabolic profiles with depth in an Atacama playa. Scheme showing the relevant biomarkers (proteins) and microorganisms detected and identified *in situ* with SOLID-LDChip (blue lettering), with ground-truth LDChip only (asterisks), metaproteomics studies in the laboratory (shadowed in yellow), and by DNA sequencing and metaproteomics (black lettered microbial names). Most of the biomarkers and microorganisms identified were related to nitrogen, sulfur, and carbon metabolisms (green shadowed boxes). Depth scale (cm) and the C/N ratio are indicated on the left, as well as a graphic indication of the overall amount of biomarkers detected with SOLID, both in number and in amount as inferred from the fluorescence intensity. See Results section 3.1 for further details.

Besides detecting signs of microbial life, SOLID-LDChip allowed us to further draw a rough microbial and metabolic vertical profile from the surface down to 80 cm (Fig. 8). SOLID-LDChip not only confirmed the higher microbial and metabolic diversity of shallower sediments (0–50 cm) but also confirmed the more specialized metabolic activity in deeper ones (50–80 cm).

SOLID-LDChip showed immunodetections of strains and proteins related to nitrogen metabolism. These include nitrogen fixers such as *Azotobacter* (Gammaproteobacteria, Pseudomonadaceae family) and nitrogen reducers/denitrifiers such as *Polaromonas*, *Dechloromonas* (Betaproteobacteria), and *Magnetospirillum* (Alphaproteobacteria) as well as the key components of the nitrogenase complex, (NifH, NifD, and NifS) nitrate reductase (NRA), nitrite oxidoreductase (NorB), or the general regulator of nitrogen metabolism (GlnB) (Fig. 8).

Metaproteomics confirmed the presence of these microorganisms and added new ones (*e.g.*, the nitrogen-fixing Alphaproteobacteria *Methylobacterium* spp., and *Rhodospirillum* spp). Similarly, metaproteomic analyses confirmed the presence of nitric oxide dioxygenase and GlnB proteins, both related to nitrogen metabolism.

The detection of markers related to nitrogen metabolism by SOLID-LDChip indicates how relevant this metabolism is in the sampling area, where low biomass results in nitrate metabolism and nitrogen fixation playing a pivotal role in the system as has recently been reported (Shen *et al.*, 2021, 2022). Although genes and proteins involved in nitrogen fixation have been found in hyperarid soils (Wilhelm *et al.*, 2018; Fernández-Martínez *et al.*, 2019; Shen *et al.*, 2021, 2022), apart from Chloroflexi, Cyanobacteria and other potential nitrogen fixers are absent or patchy present near the surface (Warren-Rhodes *et al.*, 2019); therefore, inputs due to nitrogen fixation seem to play a minor role in the nitrogen cycle (Shen *et al.*, 2022).

Further, it has been described that nitrate can be driven to denitrification or to dissimilatory nitrate reduction to ammonia (DNRA) as a function of the C/N ratio or the redox potential among other factors (van der Berg *et al.*, 2016; Huang *et al.*, 2021; Shen *et al.*, 2022). In our study, the low C/N ratios determined, as well as the trend of decreasing C/N ratio with depth (Fig. 7B), confirm previous works suggesting that nitrate reduction and nitrification pathways might be operating in certain favorable niches (Fernández-Martínez *et al.*, 2019; Shen *et al.*, 2019, 2021, 2022).

On the other hand, sulfate reducers have also been found in other playa sediments, being more abundant where sulfate concentrations are higher (Fernández-Martínez *et al.*, 2019; Warren-Rhodes *et al.*, 2019). SOLID showed the presence of biomarkers from heterotrophic Actinobacteria such as *Streptomyces* and *Frondihabitans*, as well as the Firmicutes *Sulfobacillus*, which could use reduced sulfur compounds as energy sources in microaerobic and anaerobic niches.

Altogether, the detected microorganisms would account for the production of one of the most universal proteins, the ATP synthase, which was also detected by SOLID and corroborated by metaproteomics (Fig. 3C, D and Supplementary Tables S4 and S5), and is responsible for the production of the cellular energetic currency, the ATP. Despite the presence of sulfate-reducing microorganisms, mainly Deltaproteobacteria distributed homogeneously in SR sediments, no direct signals of dissimilatory sulfite reductase (DsrAB) were detected with SOLID, probably due to the low signals obtained in all samples. However, these proteins were present in drillhole H1-H1A, where the 50–60 cm interval achieved the highest intensity values, in agreement with the higher sulfate and gypsum concentrations found in the Playa.

Recently, “islands” of habitability have been observed in the Atacama Desert. The intracellular and extracellular ATP analyses carried out by Schulze-Makuch *et al.* (2021) and the detection of living cells by microscopy (Azua-Bustos *et al.*, 2020) have proved the presence of active microorganisms in lithic environments and clays, respectively.

Other studies based on culture-dependent methods have corroborated the presence of live aerobic microorganisms at the Yungay region (Schulze-Makuch *et al.*, 2018; Shen *et al.*, 2021). Overall, some microorganisms are probably in a dormant state in sediments, and when conditions are more favorable, for example, if water content increases, they become active. Thus, Firmicutes (mainly Bacilli class), found at all depths, has been previously reported (Warren-Rhodes *et al.*, 2019) in similar samples. In addition, the presence of ATPases, cytochrome oxidase, and SHMT proteins at depths below 40–50 cm might indicate the presence of living cells or a very recent past cellular activity due to recent rain events (Schulze-Makuch *et al.*, 2018; Fernández-Martínez *et al.*, 2019).

None of the analytical techniques applied in this work can indicate whether microorganisms are active or not. However, the results confirm that there are either recent or very well-preserved microbial biopolymers and molecular biomarkers that furnish information about the biodiversity and the metabolisms operating at the cell proliferation stages, mostly during and just after wetting events (Schulze-Makuch *et al.*, 2018; Fernández-Martínez *et al.*, 2019).

The full LDChip analysis conducted *in situ* during the operation at the field-based laboratory for all ground-truth holes and performed in parallel to the robotic drills (Fig. 4) confirmed the results obtained with SOLID onboard the rover. Although we obtained additional immunodetections that were not seen with SOLID, the results agreed on a drop in the number of positive signals in the deepest sample (70–80 cm).

Overall, the biomarker pattern exhibited variation (Fig. 4), but statistically revealed greater similarities and a definite cluster within drillholes in the gypsum-rich (SR) area than with the DF samples (Supplementary Tables S1 and S2 and Supplementary Fig. S1). In general, immunodetections were more intense between 10 and 50 cm, in agreement with a higher water residence at these depths as reported elsewhere (Fernández-Martínez *et al.*, 2019; Warren-Rhodes *et al.*, 2019).

Unlike Warren-Rhodes *et al.* (2019), which indicated that sediments below 50 cm seemed to be dominated nearly exclusively by the Alphaproteobacteria (*Methylobacterium radiotolerans*), SOLID could identify low-intensity immunosignals associated with Actinobacteria and Firmicutes, which were later confirmed with the manually operated ground-truth LDChip.

Besides, the detection of Deltaproteobacteria class by the LDChip and metaproteomics (Syntrophobacterales) in the middle and bottom samples, as well as the Crenarchaeota (Sulfolobales) phylum, both with sulfur reducer members, likely indicates the presence of anoxic environments, which emphasizes how specialized the Atacama playas are regarding sulfur and nitrogen cycles (Warren-Rhodes *et al.*, 2019; Shen *et al.*, 2021).

Previous works have highlighted the difficulties to obtain-good quality DNA in hyper-arid soils: its uneven distribution, the low yields, and how the presence of salts hinders DNA extraction in deeper sediments, mostly giving negative results (Schulze-Makuch *et al.*, 2018; Shen *et al.*, 2019; Warren-Rhodes *et al.*, 2019). A number of microbial sequences found in this work were associated with the common human or animal microflora and were removed as contamination.

Although sampling was performed after an exhaustive cleaning procedure of the instrumentation, and amplicon sequences were carefully filtered to remove contaminants, we cannot rule out that a number of them were indeed there, coming from animal residues that recent years runoff has gathered at this small evaporitic basin (Supplementary Fig. S4).

It is well known that PCR amplification on poor DNA preparations can add extra biases to the final sequence diversity. These drawbacks support and encourage the use of complementary techniques such as metaproteomics and immunoassays, which are less sensitive to such bias because they are based on the whole biomass as target.

In spite of that, DNA sequences from indigenous soil microorganisms were clearly identified, confirming many SOLID-LDChip results and proving the presence of additional phylogenetic groups (Fig. 8). The presence of DNA sequences from *Frankia* spp. (Actinobacteria) and members of the Rhizobiales order (Alphaproteobacteria), both capable of nitrogen fixation in the upper samples, would explain the detection of proteins involved in nitrogen fixation by SOLID-LDChip (Figs. 3 and 4).

However, most of the proteins detected by metaproteomics were assigned to Proteobacteria, in particular to Gammaproteobacteria (25% assigned to *Pseudomonas* spp), and only a small fraction (7%) was assigned to nitrogen-fixing Rhizobiales and Rhodospirillales orders. This lack of agreement between methods at the phylogenetic level may be attributed to the bias of the protein annotation in the databases, overrepresented by *Pseudomonas* strains, or to the extraction methods that could favor Gram-negative bacteria lysis rather than Gram-positive ones (Actinobacteria and Firmicutes), which have thick cell walls.

Finally, differences in the biomarker patterns obtained between the two locations (SR and DF) by FSMI immunograms—a result also found in previous studies of playa sediments versus alluvial pavement soils elsewhere in the Atacama following rainfall events (Fernández-Martínez *et al.*, 2019; Warren-Rhodes *et al.*, 2019)—were seen not only in the relative abundance of the main phylogenetic groups but also in the proteomic profile of the biomarkers.

These variations could be explained, at least to some extent, by the differences in the geochemical composition of the samples (*i.e.*, sulfate, nitrate, or chloride content) (Fig. 7A, B), as well as the longer water residence expected in the samples within the SR basin (Meslier *et al.*, 2018; Fernández-Martínez *et al.*, 2019; Warren-Rhodes *et al.*, 2019). Thus, members of the Chloroflexi phylum (order Kallotenuales), the Acidimicrobiia class, and the order Burkholderiales were only present in DF sediments (0–20 cm), whereas the SR samples were dominated by members of the Actinobacteria and Alphaproteobacteria classes, as in other studies in the same area and with similar sample features (Warren-Rhodes *et al.*, 2019; Schulze-Makuch *et al.*, 2021). By contrast, Actinobacteria were underrepresented by metaproteomics, whereas Proteobacteria, Planctomyces, and Firmicutes were the most abundant phyla detected.

Primary producers would fix N_2_ at the top zone together with some bacteria responsible for the sulfur metabolism in the upper layers of the SR sediments, whereas heterotrophy and nitrate reduction would be heterogeneously distributed along the vertical profile. Protein markers for oxidative stress, probably as a consequence of desiccation, were also detected by SOLID-LDChip (CydA) (Fernández-Martínez *et al.*, 2019) as well as others required for the activation of the one-carbon-source utilization pathway.

On the other hand, the analysis of most functional groups of proteins revealed significant differences in the biomarker profile of the H4 samples (DF) with respect to the SR samples. Thus, the detection of Rubisco protein on the surface of the sediments in the DF was in agreement with the presence of the autotroph phylum Chloroflexi as reported (Warren-Rhodes *et al.*, 2019). With the exception of hypolithic environments, Archaea is generally absent in surface sediments (Navarro-González *et al.*, 2003; Crits-Christoph *et al.*, 2013; Azua-Bustos *et al.*, 2018; Warren-Rhodes *et al.*, 2019) or represented by only a few sequence reads (Meslier *et al.*, 2018; Schulze-Makuch *et al.*, 2018; Fernández-Martínez *et al.*, 2019). Very few DNA sequences from Archaea were obtained, with Crenarchaeota being the only phylum detected by amplicon sequencing in the upper fraction of the SR sediments (members of the ammonia-oxidizing Nitrososphaerales), particularly in sample S-H2b (10–20 cm).

However, members of Euryarchaeota (Methanobacteriales, Haloferacales, and Methanosarcinales) and Crenarchaeota were identified by metaproteomics in the top and bottom pools (Fig. 6) and by manually operated LDChip analysis in the lab (Fig. 4), in agreement with Fernández-Martínez *et al.* (2019) findings. Surprisingly, a thermophilic Archaea and *Halorubrum* (Haloarchaea) were identified in all depths for hole H2 in ground-truth LDChip analysis (Fig. 4).

These substantial discrepancies found among the Archaea community are likely associated with the different methods applied, and particularly when comparing LDChip results with amplicon sequencing data. Primarily, the discrepancies can be attributed to the scarce amount of sample available after sample delivery into the instruments, since sequencing performance is limited when a small sample or number of cells is available (Lombard *et al.*, 2011). Further, the low prokaryotic community biomass frequently associated with hyperarid soils also results in low amounts of DNA in sediments, such as those from Playa-like sites, which ultimately can lead to an underestimation of the microbial community (Schulze-Makuch *et al.*, 2018; Warren-Rhodes *et al.*, 2019).

### 4.3. Metaproteomics corroborated and further characterized the microbial and metabolic profiles

Metaproteomics has proven its high potential for documenting microbial diversity, profiling metabolic function, and elucidating the ecological role of microorganisms in diverse environments (Liu *et al.*, 2016; Wang *et al.*, 2016; Pjevac *et al.*, 2018; Martínez-Alonso *et al.*, 2019; Maseh *et al.*, 2021). To date, however, only a few metaproteomic studies have documented the subsurface protein profile of the hyperarid core of the Atacama Desert (Bull *et al.*, 2016; Schulze-Makuch *et al.*, 2018; Fernández-Martínez *et al.*, 2019).

Despite the scarce amount of organic matter described, those studies obtained valuable information. In our current study, the TOC content of the Playa samples was lower than that reported previously in sediments from the same area (Warren-Rhodes *et al.*, 2019). However, both sets of data share the same depth-dependent pattern, with higher values in the top 20 cm than in the 50–80 cm (Fig. 7B). Similarly, metaproteomics revealed higher quantities of proteins in the upper 50 cm, which suggests a higher metabolic activity in these (0–50 cm) versus deeper layers (50–80 cm). This is particularly relevant for proteins related to nitrogen metabolism, as has been previously reported for gypsum-rich sediments (López-Lozano *et al.*, 2012).

The δ^15^N values in top and middle pool samples (0–50 cm) were slightly positive (Fig. 7B), and thus consistent with nitrogen fixation (Robinson, 2001). Consistently, Nif-like proteins were identified through metaproteomics, but nitrogen fixation activities were only detected with the SOLID-LDChip immunoassay (NifD, NifH and NifS proteins).

Hence, nitrogen fixation metabolism is likely present in the upper layers of SR sediments together with proteins involved in sulfate-reducing metabolism as detected by immunoassays (dissimilatory sulfite reductase DsrB) and metaproteomics (phosphate-binding protein PstS), associated with members of the Gammaproteobacteria class and Sulfolobales order (within the Crenarchaeota phylum) respectively.

Biomarkers related to nitrogen fixation in the surface of other playas at the Atacama Desert have been previously reported (*e.g.*, NifD and NifH proteins) (López-Lozano *et al.*, 2012; Wilhelm *et al.*, 2018; Fernández-Martínez, *et al.*, 2019;) to be mainly associated with Cyanobacteria (hypolithic and endolithic forms) as well as the Alphaproteobacteria class, which predominate in surface sediments (Meslier *et al.*, 2018).

Other nitrogen fixers broadly distributed in fertile soils (*e.g.*, *Frankia*, *Sinorhizobium*, *Rhizobium*, and *Azospirillum*) have also been detected in hyperarid soils (Connon *et al.*, 2007; Nielson *et al.*, 2017). In contrast, genes involved in nitrogen fixation (*nif* genes) have not been identified in endolithic communities (Crits-Christoph *et al.*, 2016; Finstad *et al.*, 2016).

These findings, together with the absence of measurable amounts of ammonium and the low proportion of nitrogenase detected in Atacama surface soils (Shen *et al.*, 2022), suggest that nitrogen fixation can represent a small fraction of the nitrogen cycle in gypsum soils, which is dominated by dissimilatory nitrogen reduction and nitrification pathways (Fig. 8) (López-Lozano *et al.*, 2012; Mandakovic *et al.*, 2018; Shen *et al.*, 2019, 2021, 2022).

GlnB, a nitrogen regulatory protein P-II, detected here by metaproteomics and SOLID-LDChip, is also responsible for regulating the synthesis of GS in response to nitrogen deprivation (Bueno *et al.*, 1985). With depth, nitrogen starvation appears to lead to ammonium production via alternative metabolic pathways for the synthesis of proteins and nucleic acids, and this compound can be subsequently converted to glutamine by the GS enzyme that regulates nitrogen metabolism via ammonium assimilation from different sources (Cabello *et al.*, 2004; Harper *et al.*, 2010; Bolay *et al.*, 2018).

These findings, together with the detection of proteins related with a potential nitrate assimilation metabolism (Fernández-Martínez *et al.*, 2019), support the idea that nitrate reduction seems to be a relevant metabolism in the sampling area (Shen *et al.*, 2022). Other enzymes such as the PdxS, important in the synthesis of essential cofactors, together with SHMT and GCS H protein, participate in the one-carbon metabolism, a complex network of folate-dependent chemical reactions to build essential biomolecules, including amino acids such as methionine, as well as nucleotides for DNA synthesis, necessary for cell growth (Sodolescu *et al.*, 2018).

This type of metabolism has previously been described for *M. radiotolerans*, which seem to dominate these oligotrophic subsurface sediments (Warren-Rhodes *et al.*, 2019). In addition, a nitric oxide dioxygenase protein involved in the denitrification pathway was detected in the bottom pool sample (50–80 cm), probably in response to low levels of oxygen and low carbon content. This pathway might serve as an alternative pathway for ammonium production, enhancing NH_4_^+^ availability and uptake and contributing to nitrogen retention (Shen *et al.*, 2019).

This limiting oxygen availability might explain the detection not only of proteins assigned to methanogenic archaea by metaproteomics such as a phosphonate-transporting ATPase protein (*Methanobacterium*) associated with low phosphate environments (Vikran *et al.*, 2016) but also the methyl CoA reductase (McrB) protein by the SOLID-LDChip in holes S-H1A and H3 (below 50 cm) (Schulze-Makuch *et al.*, 2018; Fernández-Martínez *et al.*, 2019).

In turn, these activities could provide a carbon source for a methylotrophic community made up of members of orders Rhizobiales, Caulobacterales, and Pseudomonadales (Schulze-Makuch *et al.*, 2021).

Geochemical parameters and environmental conditions described for the Playa area undoubtedly suggest nutrient limitation and stress conditions that require most of the microbial population to persist primarily in a dormant state. We have previously reported stress protein profiles by the LDChip as indicators of basal metabolic activity (Schulze-Makuch *et al.*, 2018; Wilhelm *et al.*, 2018; Fernández-Martínez *et al.*, 2019), which provided evidence that the driest Atacama surface soils represent a threshold for long-term habitability (*i.e.*, growth and reproduction).

In this study, proteins related to oxidative stress, which are associated with carbon and nitrogen limitations, were detected by remote SOLID, in-ground-truth analysis (S-H1Ae) with LDChip in the lab, and by metaproteomic analysis. The cytochrome bd-type ubiquinol oxidase (CydA), for example, is widely distributed in prokaryotes and predominates under microaerobic conditions, playing a role in aerotolerant nitrogen fixation and protection against metal toxicity and oxidative stress in *Azotobacter* species (Poole, 1994).

## 5. Conclusions

We demonstrated a successful remote life detection and characterization experiment with the SOLID-LDChip instrument in a Mars drilling simulation campaign to the hyperarid core of the Atacama Desert. We detected molecular biomarkers along a vertical profile down to 80 cm and inferred relevant metabolisms that might have been critical at the time of sampling or sometime before.

With SOLID results only, we detected changes in the bacterial community and metabolisms with depth in the evaporitic playa. Sulfate and nitrate reducing bacteria and, to a lesser extent, nitrogen-fixing bacteria seem to dominate the system and provide organic matter to sustain the heterotrophic community. SOLID results were validated through a comprehensive ground-truth, multi-approach analysis on the lab combining LDChip immunoassays, DNA phylogenetic analysis, and metaproteomic studies.

With the analysis performed herein, we cannot state with total certainty whether all the microorganisms and biomarkers identified in this evaporitic playa are autochthonous or allochthonous (transported with the wind or by run-off of sporadic rain events). The findings show a distribution pattern with depth that could be associated with the water availability, C/N ratio, or the specific local geochemistry that can change within only a few meters apart (Fig. 7).

The detection of proteins such as ATP synthase, preferably in the top layers, together with proteins involved in oxidative and hydric stress (CydA, GS or PdxS), is consistent with current or recent metabolic activity under stress. Proteins and microorganisms capable of nitrogen fixation coexist with nitrate reducers that dominate the upper parts, being the primary producers of the system, whereas in the lower parts, the denitrification processes seemed to dominate. The fact that SOLID detected spores, autochthonous or allochthonous, from Actinobacteria, indicates that they could germinate under favorable conditions and then become part of the local community.

The proteomic data indicate differences in the relative proportion between Bacteria, Archaea, and Eukaryota, with depth, besides significant differences in the protein patterns and their cellular metabolic associations (Fig. 6). Altogether, the data indicate that, indeed, most of the microbial community we are describing is the result of autochthonous metabolic activity produced during and after wet events.

A similar conclusion was drawn in other studies performed after rain events in which actual DNA replication was demonstrated (Schulze-Makuch *et al.*, 2018). However, if it were just the result of biomass accumulation, slow death, and degradation, a similar and smoothly changing pattern with depth would be expected, which does not seem to be the case.

Further, the differences found in the microbial composition between both types of sediments analyzed (DF vs SR) suggest that the geochemical composition, with a higher concentration of chloride, nitrate, and sulfate, as well as the orographic situation of SR out of the accumulation part of the playa, may be main factors driving the microbial community composition.

## Supplementary Material

Supplemental data

Supplemental data

Supplemental data

Supplemental data

Supplemental data

Supplemental data

Supplemental data

Supplemental data

Supplemental data

Supplemental data

## Abbreviations Used

ARADS: Atacama Rover Astrobiology Drilling Studies
ASV: amplicon sequence variant
COGs: Clusters of Orthologous Groups of proteins
DF: desert floor
EC: extraction cell
EPS: exopolymeric substances
FSMI: fluorescence sandwich microarray immunoassay
GS: glutamine synthetase
IC: ion chromatography
IMI/CMI: inhibition microarray inmunoassay/competitive microarray immunoassay
IRMS: isotope-ratio mass spectrometry
KEGG: Kyoto Encyclopedia of Genes and Genomes
LDChip: Life Detector Chip
LOD: limit of detection
MAAM: multiarray analysis module
MS/MS: tandem mass spectrometry
NC: negative control
NSAF: normalized to spectral abundance factor
PAH: polycyclic aromatic hydrocarbon
PCA: principal components analysis
PCR: polymerase chain reaction
PdxS: pyridoxal 5′-phosphate synthase subunit
rRNA: ribosomal RNA
SAU: sample analysis unit
SDS-TCA: sodium dodecyl sulfate-trichloroacetic acid
SHMT: serine hydroxymethyltransferase
SOLID: Signs of Life Detector
SPU: sample preparation unit
SR: sulfate-rich
TN: total nitrogen
TOC: total organic carbon
USGS: United States Geological Survey
UV: ultraviolet
XRD: X-ray diffraction

## Notes

Associate Editor: Don Cowan
