# Supplementary material for: Life Detection and Microbial Biomarker Profiling with Signs of Life Detector-Life Detector Chip During a Mars Drilling Simulation Campaign in the Hyperarid Core of the Atacama Desert
Source: Astrobiology. 2023 Dec 20;23(12):1259–83. doi: 10.1089/ast.2021.0174 (PMC10825288; doi:10.1089/ast.2021.0174)
Supplement: Supplemental data [file Suppl_FigS4.docx]

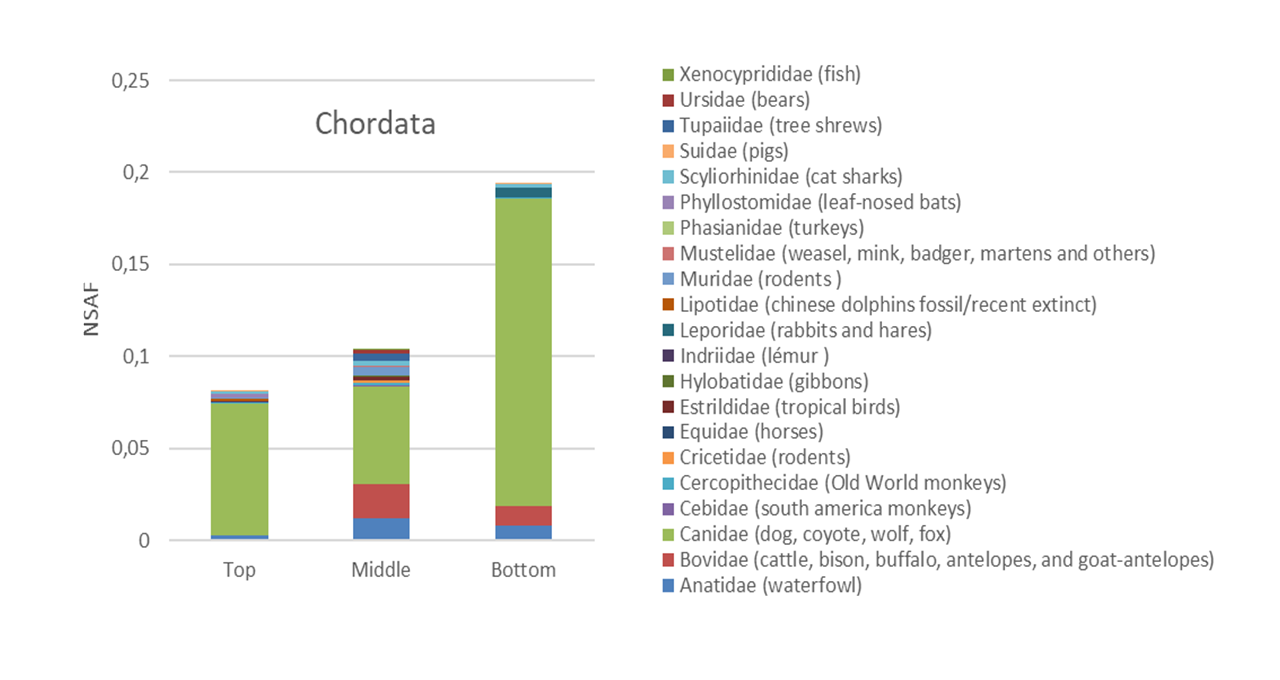


**Figure S4.** Phylogenetic distribution of Chordata proteins in SR sediments (top, middle and bottom samples) based on the metaproteomic analysis, as relative abundance of normalized spectral abundance factor (NSAF).
