# Supplementary material for: Life Detection and Microbial Biomarker Profiling with Signs of Life Detector-Life Detector Chip During a Mars Drilling Simulation Campaign in the Hyperarid Core of the Atacama Desert
Source: Astrobiology. 2023 Dec 20;23(12):1259–83. doi: 10.1089/ast.2021.0174 (PMC10825288; doi:10.1089/ast.2021.0174)
Supplement: Supplemental data [file Suppl_TableS1.docx]

**Table S1: Pairwise linear correlation *r* between the four cores H1, H2, H3 and H4.**

|  | **H1** | **H2** | **H3** | **H4** |
| --- | --- | --- | --- | --- |
| **H1** |  | 2.05E-01 | 4.39E-01 | 1.10E-01 |
| **H2** | 2.05E-01 |  | 2.75E-01 | 3.31E-02 |
| **H3** | 4.39E-01 | 2.75E-01 |  | 2.66E-01 |
| **H4** | 1.10E-01 | 3.31E-02 | 2.66E-01 |  |
|  |  |  |  |  |
