# Supplementary material for: Life Detection and Microbial Biomarker Profiling with Signs of Life Detector-Life Detector Chip During a Mars Drilling Simulation Campaign in the Hyperarid Core of the Atacama Desert
Source: Astrobiology. 2023 Dec 20;23(12):1259–83. doi: 10.1089/ast.2021.0174 (PMC10825288; doi:10.1089/ast.2021.0174)
Supplement: Supplemental data [file Suppl_TableS2.docx]

**Table S2: P-values corresponding to the pairwise linear correlations *r* shown in Table S1**. Note that if the p-value is small (e.g. p-value<0.05), the correlation *r* in Table S1 is significantly different from zero.

|  |  |  |  |  |
| --- | --- | --- | --- | --- |
|  | **H1** | **H2** | **H3** | **H4** |
| **H1** |  | 1.18E-02 | 1.95E-08 | 1.80E-01 |
| **H2** | 1.18E-02 |  | 6.63E-04 | 6.88E-01 |
| **H3** | 1.95E-08 | 6.63E-04 |  | 1.02E-03 |
| **H4** | 1.80E-01 | 6.88E-01 | 1.02E-03 |  |
