# Supplementary material for: Life Detection and Microbial Biomarker Profiling with Signs of Life Detector-Life Detector Chip During a Mars Drilling Simulation Campaign in the Hyperarid Core of the Atacama Desert
Source: Astrobiology. 2023 Dec 20;23(12):1259–83. doi: 10.1089/ast.2021.0174 (PMC10825288; doi:10.1089/ast.2021.0174)
Supplement: Supplemental data [file Suppl_FigS1.docx]

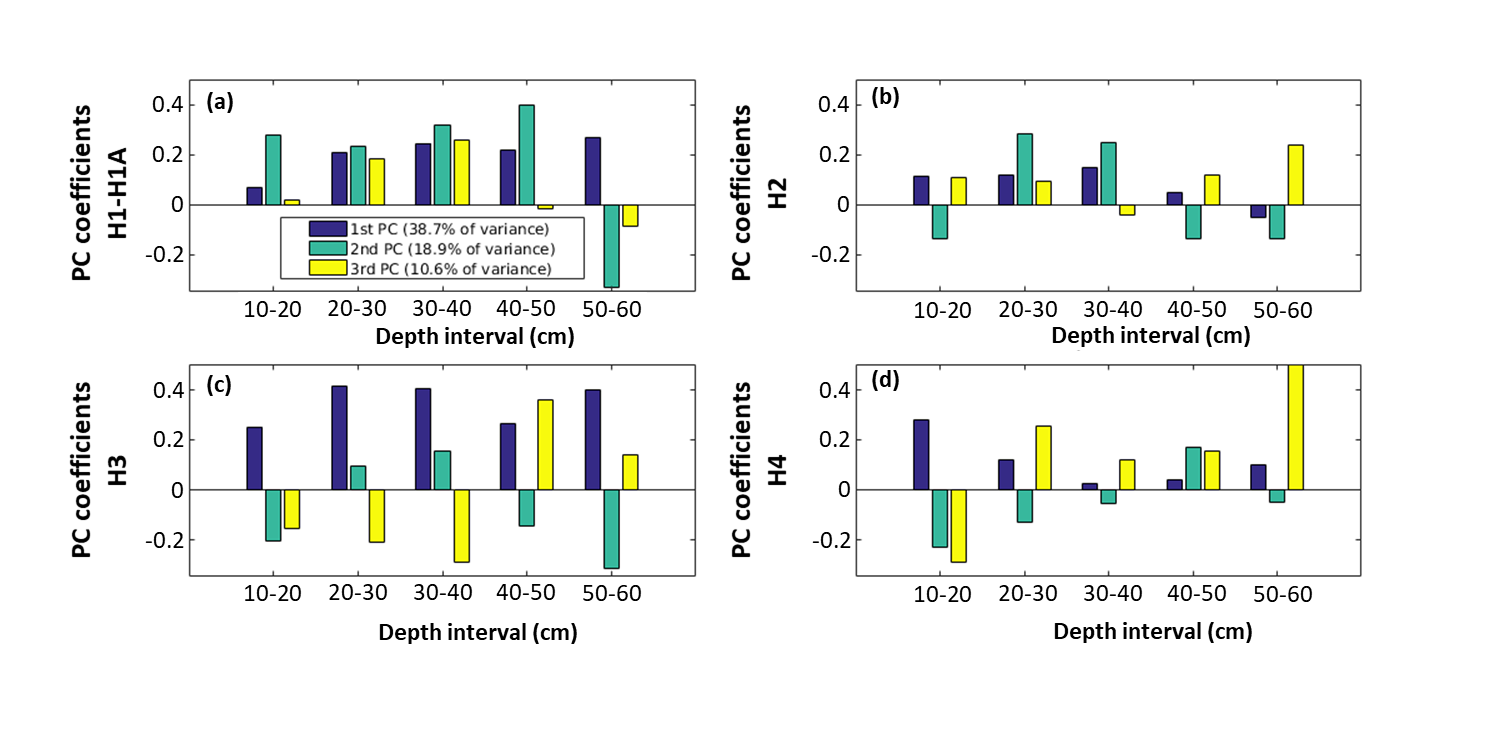


**Figure S1:** **Principal Components Analysis (PCA) of the population abundances measured at the four cores and for depths from 10 to 60 cm based on LDCHip immunoassays results.** The 1^st^, 2^nd^ and 3^rd^ principal components coefficients are plotted, which explain 39%, 19% and 11% of the total variance respectively. For clarity, results are split in four different plots: (a) H1, (b) H2, (c) H3 and (d) H4.
