# Supplementary material for: Life Detection and Microbial Biomarker Profiling with Signs of Life Detector-Life Detector Chip During a Mars Drilling Simulation Campaign in the Hyperarid Core of the Atacama Desert
Source: Astrobiology. 2023 Dec 20;23(12):1259–83. doi: 10.1089/ast.2021.0174 (PMC10825288; doi:10.1089/ast.2021.0174)
Supplement: Supplemental data [file Suppl_FigS2.docx]

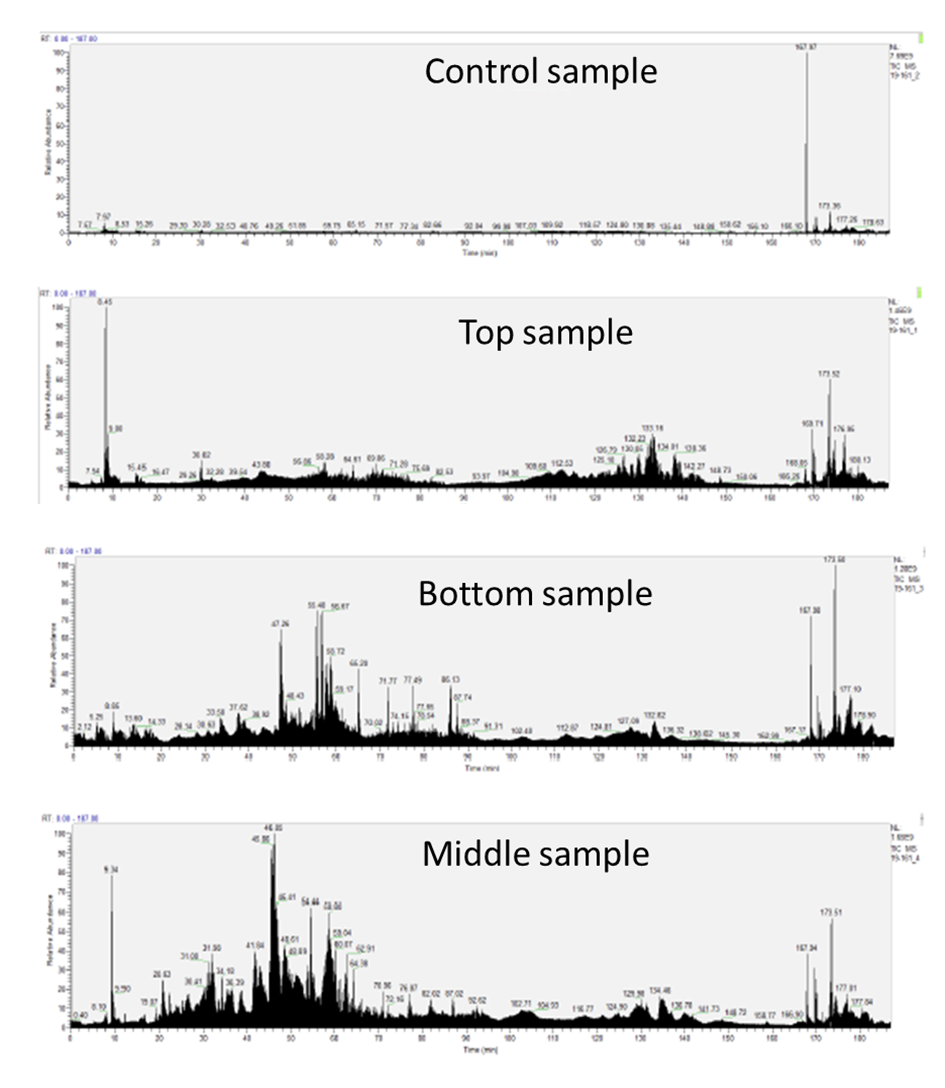


**Figure S2.** Metaproteomic sequencing chromatograms obtained with three (top, middle and bottom) samples and an extraction control sample (only buffers) by LC-MS/MS on a Q-Exactive HF mass spectrometer (Thermo) in a data-dependent acquisition (DDA) mode. Analyses were performed at the Proteomics Unit of the Complutense University of Madrid.
