# Supplementary material for: Life Detection and Microbial Biomarker Profiling with Signs of Life Detector-Life Detector Chip During a Mars Drilling Simulation Campaign in the Hyperarid Core of the Atacama Desert
Source: Astrobiology. 2023 Dec 20;23(12):1259–83. doi: 10.1089/ast.2021.0174 (PMC10825288; doi:10.1089/ast.2021.0174)
Supplement: Supplemental data [file Suppl_TableS4.docx]

**Table S4.** Values of NSAF of prokaryotic proteins identified in top (H1A drill, 10-20 cm), middle (H1A, 20-50 cm) and bottom (H3S1, 40-80 cm) pool of samples. NSAF refers to the normalized spectral abundance factor.
