# Supplementary material for: Life Detection and Microbial Biomarker Profiling with Signs of Life Detector-Life Detector Chip During a Mars Drilling Simulation Campaign in the Hyperarid Core of the Atacama Desert
Source: Astrobiology. 2023 Dec 20;23(12):1259–83. doi: 10.1089/ast.2021.0174 (PMC10825288; doi:10.1089/ast.2021.0174)
Supplement: Supplemental data [file Suppl_TableS3.docx]

| **Table S3.** Richness based on ASV (Amplicon sequence variants), Shannon–Wiener (H') Diversity, and Evennes indices from the High-Throughput Sequencing data of the Bacterial 16S rRNA Genes. | | | | | | | | |
| --- | --- | --- | --- | --- | --- | --- | --- | --- |
|  |  | |  |  |  |  |  |  |
| **Sample** | | **Depth intervals** | **Sequences** | **Nº of sequences** | **Abundance** | **Rinchnes** | **Shannon (H')** | **Evennes** |
|  | | **(cm)** | **generated** | **after processing** |  | **( number of ASVs)** |  |  |
|  | |  |  |  |  |  |  |  |
|  | |  |  |  |  |  |  |  |
| S-H1b | | 10-20 | 164,428 | 68,556 | 10,051 | 9 | 1.88 | 0.73 |
| S-H2b | | 10-20 | 261,446 | 85,078 | 7,373 | 2 | 0.70 | 1.0 |
| S-H3d | | 30-40 | 211,739 | 89,595 | 23,230 | 12 | 2.13 | 0.70 |
| S-H1Ae | | 40-50 | 163,183 | 63,727 | 1,556 | 1 | 0 | 1.0 |
| S-H4b | | 10-20 | 255,773 | 77,588 | 35,833 | 54 | 2,84 | 0.32 |
|  | |  |  |  |  |  |  |  |
