# Supplementary material for: Life Detection and Microbial Biomarker Profiling with Signs of Life Detector-Life Detector Chip During a Mars Drilling Simulation Campaign in the Hyperarid Core of the Atacama Desert
Source: Astrobiology. 2023 Dec 20;23(12):1259–83. doi: 10.1089/ast.2021.0174 (PMC10825288; doi:10.1089/ast.2021.0174)
Supplement: Supplemental data [file Suppl_FigS5.docx]

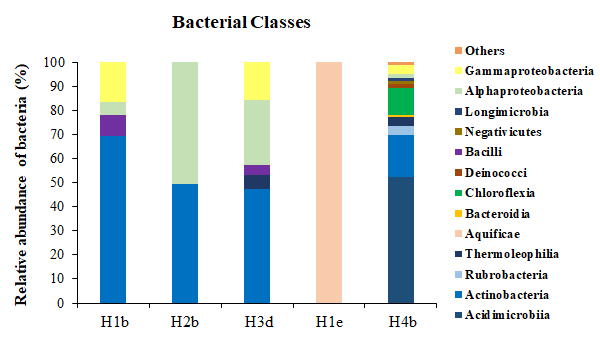


Figure S5. Bacterial composition at the class level of SR sediments (Samples S-H1b, SH2b, S-H3d and S-H1Ae) and DF sediments (sample S-H4b) from the 16 rDNA gene sequencing from samples delivered to instruments. Only bacterial classes accounting for >1% of total number of sequences are plotted~~.~~
